# Enhanced proteomic profiling of human plasma‐derived extracellular vesicles through charge‐based fractionation to advance biomarker discovery potential

**DOI:** 10.1002/jev2.70024

**Published:** 2024-12-06

**Authors:** Xianyi Su, Getúlio Pereira de Oliveira Júnior, Anne‐Lise Marie, Michal Gregus, Amanda Figueroa‐Navedo, Ionita C. Ghiran, Alexander R. Ivanov

**Affiliations:** ^1^ Department of Chemistry and Chemical Biology, Barnett Institute of Chemical and Biological Analysis Northeastern University Boston Massachusetts USA; ^2^ Department of Anesthesia, Beth Israel Deaconess Medical Center Harvard Medical School Boston Massachusetts USA

**Keywords:** EV sub‐populations, extracellular vesicles, ion exchange chromatography, prostate cancer diagnostics, proteomics

## Abstract

The study introduces a charge‐based fractionation method for fractionating plasma‐derived extracellular vesicles (EVs) into sub‐populations aimed at the improved purification from free plasma proteins to enhance the diagnostic potential of EV sub‐populations for specific pathophysiological states. Here, we present a novel approach for EV fractionation that leverages EVs’ inherent surface charges, differentiating them from other plasma components and, thus, reducing the sample complexity and increasing the purity of EVs. The developed method was optimized and thoroughly evaluated using proteomic analysis, transmission electron microscopy, nanoparticle tracking, and western blotting of isolated EVs from healthy donors. Subsequently, we pilot‐tested the developed technique for its applicability to real‐world specimens using a small set of clinical prostate cancer samples and matched controls. The presented technique demonstrates the effective isolation and fractionation of EV sub‐populations based on their surface charge, which may potentially help enhance EV‐based diagnostics, biomarker discovery, and basic biology research. The method is designed to be straightforward, scalable, easy‐to‐use, and it does not require specialized skills or equipment.

## 1 INTRODUCTION

Extracellular vesicles (EVs) are nanometre‐scale, phospholipid bilayer membrane‐enclosed globular entities actively secreted into the extracellular milieu by various cell types (Théry et al., 2018; Welsh et al., 2024). Non‐vesicle extracellular particles (NVEPs) are similar to EVs in their low‐nanometre size ranges and representation of molecular types, while they lack the lipid bilayer membrane and the vesicular morphology (Welsh et al., 2024). For simplicity, here, we use the term EVs to combine both EVs and particles. EVs function as pivotal cellular messengers by transporting a diverse assortment of bioactive cargo, including proteins, lipids, glycans, and nucleic acids (Théry et al., 2018). Given their significant roles in intercellular communication and their involvement in physiological and pathological processes (Van Niel et al., 2018), EVs have gained considerable attention as promising candidates for molecular characterization. In the context of discovery proteomic research, while liquid chromatography–tandem mass spectrometry (LC‐MS/MS) has been a mainstay in analysing the complex proteome composition of EVs, it faces significant challenges in sensitivity, specificity, quantitative accuracy, and dynamic range (both at the sample matrix level and within the EV proteome), particularly when dealing with the diverse and often minute biomolecular content of these vesicles originated from complex source matrices (Gandham et al., 2020).

Among the various types of EVs of different origins, circulating EVs, specifically those derived from plasma, present a unique opportunity for minimally invasive biomarker discovery (Szatanek et al., 2015). These vesicles act as surrogates of their parent cells, being easily obtained from plasma and other biofluids. This is advantageous compared to collecting tissue samples that require more invasive procedures. Proteomic profiling of plasma‐derived EVs may offer a more consistent and effective approach for biomarker identification over conventional plasma proteomics, which is often compromised by a high dynamic range and high‐abundance‐free plasma proteins (Kaur et al., 2021). Notwithstanding these advantages, the isolation and characterization of circulating EVs face significant technical and methodological challenges. Widely used isolation methods such as ultracentrifugation and size exclusion chromatography (SEC) encounter difficulties in separating high‐abundance free plasma proteins/protein complexes from EVs, which affects the accurate identification and quantification of EV‐specific biomolecules (Taylor & Shah, 2015). Furthermore, these methods fall short in one additional aspect: the ability to fractionate EVs into sub‐populations. Such fractionation is vital for a deeper understanding of EVs, as these sub‐populations can exhibit unique characteristics that are indicative of their potential role in physiological or pathological processes.

Recent studies have explored various fractionation methods to fill these needs. For example, heparin affinity‐based fractionation has been utilized to separate EV sub‐populations effectively, though this technique was primarily used for the fractionation of EVs from less complex biomatrices than plasma (Zhou et al., 2023). Similarly, charge‐based fractionation methods using salt‐based elution have been reported (Heath et al., 2018), but their application to plasma‐derived EVs remains limited. In light of these previous studies, we propose a novel chromatography‐based EV isolation approach that exploits the unique electrostatic properties of plasma‐derived EVs. We hypothesized that a charge‐based fractionation method could reduce contamination with high‐abundance plasma proteins. Additionally, this approach aims to facilitate the fractionation of plasma‐derived EVs into distinct surface charge‐based EV sub‐populations, an approach not yet explored by existing methodologies. Our approach is grounded in two inherent characteristics of EVs. First, the phosphate head groups of the phospholipids in the outer layer of the EV membrane possess negative charges that separate them from less charged plasma species, reducing the dynamic range and content complexity. Second, varying physiological or pathological conditions can make differences in surface glycocalyx (glycoproteins (Marie et al., 2023; Marie et al., 2021), proteoglycans, glycolipids, etc.) and protein phosphorylation states, contributing to disparate charge densities and hence driving the fractionation of EV sub‐populations. This differentiation in EV sub‐populations originating at distinct biological and disease states would enhance the specificity and applicability of EV‐based diagnostics.

**FIGURE 1:**
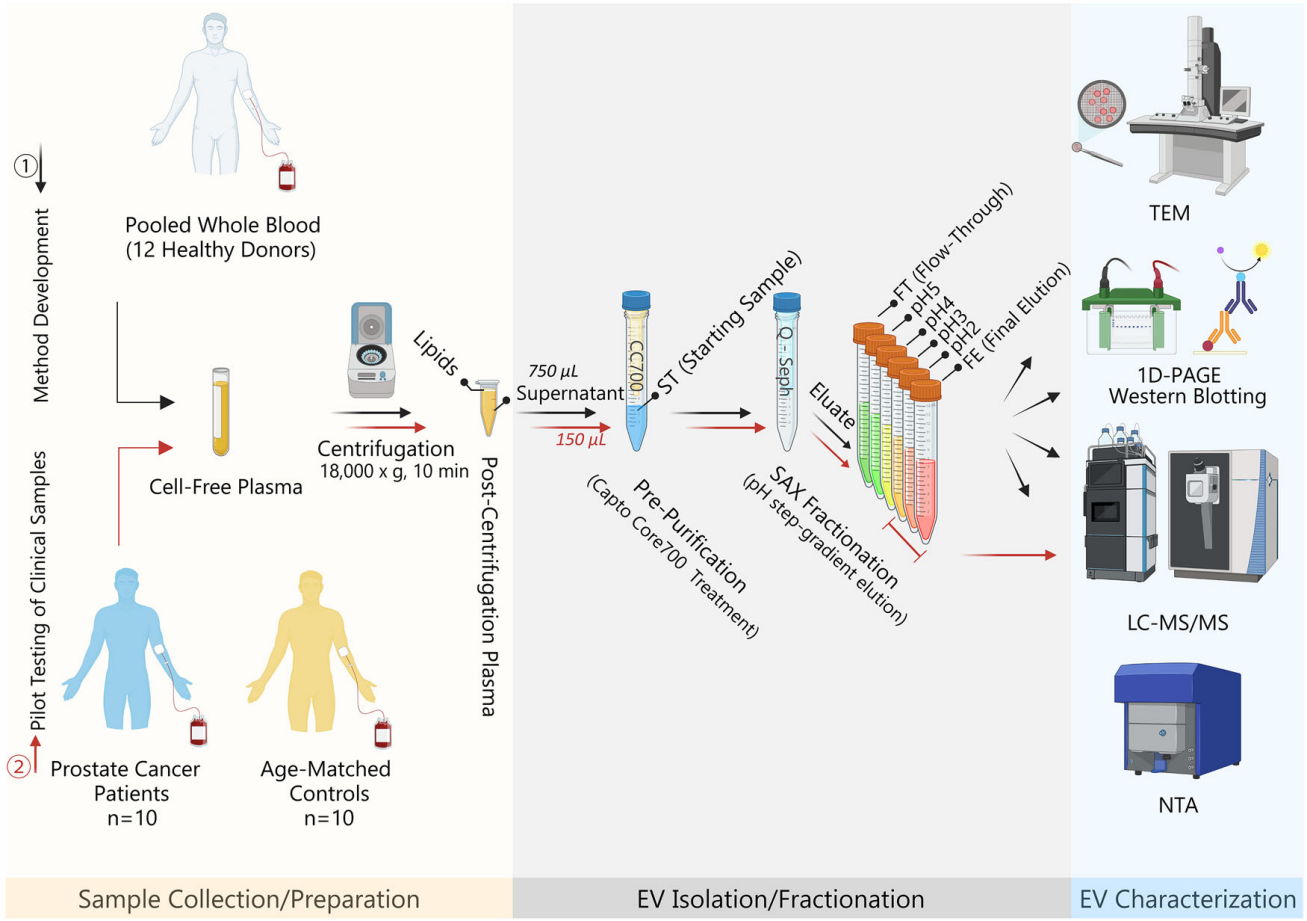
Workflow illustration. This schematic branches into two consecutive stages: ① Method development and optimization (following black arrows) and ② pilot testing of clinical samples (following red arrows). Both stages begin with whole blood collection from donors, followed by cell‐ and platelet‐free plasma preparation. An additional centrifugation step at 18,000 × *g* for 10 min is employed to mitigate lipid interference. Post‐centrifugation, the lower part of the supernatant plasma is carefully aspirated and introduced into the equilibrated Capto Core 700 (CC700) resin (resin:plasma = 3.0 mg/µL) within a spin column, followed by a 30‐min incubation on a rotating mixer. The eluate, designated as the Starting (ST) sample, is then collected in a Falcon tube after spinning down. Subsequently, the ST sample is added to equilibrated Q‐Sepharose resin (resin:plasma = 8.0 mg/µL) in a spin column, and a series of step‐gradient pH buffer elutions generates six eluate fractions, designated as flow‐through (FT), pH5, pH4, pH3, pH2, and final elution (FE) fractions. All seven samples from the method development stage undergo LC‐MS/MS‐based proteomic analysis, supplemented by orthogonal techniques, including TEM, western blotting, and NTA. Conversely, in the phase of pilot analyses of clinical samples, only the EV‐enriched fractions (i.e., the pH3, pH2 and FE fractions) are subjected to proteomic analysis.

Our study employs a two‐tiered approach to test this hypothesis. Initially, we established the charge‐based isolation method for EVs using plasma samples from self‐declared healthy donors. Subsequently, we assessed its effectiveness using plasma samples collected from prostate cancer (PCa) patients and age‐matched healthy controls (Figure 1). The selection of PCa as an exemplary focus for this study is guided by several considerations. First, PCa is a prevalent cancer type in men. According to the American Cancer Society, there will be 299,010 new PCa cases and 35,250 deaths in the United States in 2024 (Siegel et al., 2024). Additionally, PCa is known to induce specific alterations in circulating EV profiles (Vlaeminck‐Guillem, 2018), making it an appropriate case for assessing the efficiency of our charge‐based isolation method.

In the method development, optimization studies, and sub‐population enrichment validation, we implemented orthogonal techniques, including transmission electron microscopy (TEM), nanoparticle tracking analysis (NTA), and western blotting, in addition to LC–MS/MS‐based proteomic analysis for a comprehensive EV characterization and method efficiency evaluation. Next, with an established method at the pilot testing stage for clinical samples, we narrowed our focus to LC–MS/MS proteomics to explore the potential applicability of the technique in clinical diagnostics. We identified 39 differentially abundant proteins (DAPs) between the PCa and control groups, eight of which were further corroborated by the findings generated using transcriptomics techniques and reported in The Cancer Genome Atlas (TCGA) and research publications.

This study was focused on the development of a straightforward, easy‐to‐use and robust method for EV isolation and fractionation based on EVs’ surface charge. Our approach will provide the EV community with a means for uncovering and characterizing a novel charge profile dimension of circulating EVs, which could be instrumental in refining diagnostic and therapeutic strategies for PCa and potentially a range of other cancers and pathological conditions.

## 2 MATERIALS AND METHODS

For each experiment throughout this study, we calculated the volume of the samples based on the volume of plasma input and the dilution factors. The volume of input plasma is used for normalization and comparison. We chose to normalize and compare results based on the plasma input volume rather than total protein amount or EV count, as total protein assays can be influenced by non‐protein components in plasma and compounds added into the sample during EV isolation and sample processing, and EV count measurements typically lack specificity. For ease of reference and brevity, gene names are used to represent the corresponding proteins in this article (Table S1).

### 2.1 Sample collection and preparation

For method development, plasma samples were obtained from 12 self‐reported healthy male donors, aged 23–67, following IRB protocols IRB#2001P000591 (BIDMC) and IRB#17‐12‐14 (NU). Informed consent was secured from all donors. ethylenediaminetetraacetic acid (EDTA) was used as the anticoagulant for blood collection, which was then pooled to form a representative healthy donor sample. Aliquots of 1 mL from this sample were cryopreserved at −80°C. Prior to EV isolation, these samples were thawed at 37°C and centrifuged at 18,000 × *g* for 10 min, targeting a reduction in lipid interference. The lower half of the plasma supernatant was carefully aspirated, avoiding the lipid‐rich layer floating on the surface that formed post‐centrifugation.

For clinical pilot testing, plasma samples from ten PCa patients (D_01 to D_10) and 10 age‐matched healthy controls (C_01 to C_10) were obtained from Lee BioSolutions (Maryland Heights, MO). An age stratification was applied with the selection of four ranges, namely ≤60 (Groups 1 and 2), 60–70 (Groups 3–5), 70–80 (Groups 6–9), and >80 (Group 10) years‐old, respectively, to ensure varied age representation (Table S2). These samples underwent identical pre‐processing steps as the healthy donor samples, ensuring analytical consistency across the study.

### 2.2 EV isolation and fractionation

#### 2.2.1 Initial purification with Capto Core 700

In the initial purification phase, we utilized a dual‐functional chromatographic resin, Capto Core 700 (CC700), that possesses both size exclusion (molecular weight cut‐off (MWCO) ≈ 700 kDa) and anion exchange capabilities. The resin was loaded into a 5‐mL centrifuge column with a bottom frit. To ensure reproducibility, an accurate measurement of the resin dry mass, free from storage solution, was performed. This was achieved by weighing the empty column prior to its filling with resin. Subsequently, the column containing the resin was weighed after the removal of the storage solution by centrifugation at 4500 × *g* for 2 min. The dry mass of the resin was then determined by calculating the difference between the two weights. A 3.0‐mg/µL resin‐to‐plasma ratio was applied in this step (Figure S1A).

For method development, we used 750 µL of post‐centrifugation plasma from pooled healthy donors. In clinical testing, only 150 µL of plasma was required from each patient or control sample. Prior to sample introduction, the resin was equilibrated with 0.1× Dulbecco's phosphate‐buffered saline (dPBS) in a volume quadruple that of the plasma, ensuring a non‐viscous mixture consistency and maximizing binding capacity. After a 30‐min incubation at room temperature on a rotating mixer (Barnstead, USA), the mixture was centrifuged at 4500 × *g* for 5 min. The resulting solution, designated as ‘ST’ (starting sample), was meanwhile collected in a Falcon tube (plasma dilution factor = 5).

#### 2.2.2 Fractionation using Q‐Sepharose

During the fractionation phase, we packed Q‐Sepharose strong anion exchange (SAX) resin in a 5‐mL centrifuge column with a bottom frit. For method development, we processed half of the ST sample, equating to 375 µL of plasma input, with the remaining half serving as a comparative sample. For clinical testing, the entire 150 µL of plasma input volume from the ST was used. An 8.0‐mg/µL resin‐to‐plasma ratio was adopted (Figure S1B).

The fractionation process commenced by introducing the ST sample into the Q‐Sepharose resin, previously pre‐conditioned with 0.1× dPBS at a volume five times the plasma input. After collecting the flow‐through (FT) by centrifugation at 4500 × *g* for 3 min, we conducted a series of stepwise elutions with buffers at decreasing pH values (Table S3). Each elution involved a two‐step process to ensure the efficient elution of bound analytes at the target pH and appropriate equilibration of the stationary phase at a specific pH, each step involving centrifugation at 4500 × *g* for 3 min, using buffer volumes five times the plasma input, resulting in a total of 10 times the plasma input volume for each fraction. The fractions were designated as ‘FT’, ‘ pH5’, ‘pH4’, ‘pH3’, ‘pH2’, and ‘FE’ (final elution), each collected in a 10× volume in relation to the used input plasma volume.

The processed samples were then stored at −80°C until downstream analyses. However, for biophysical characterization with TEM and NTA, an immediate buffer exchange to 1× dPBS was performed. This step was crucial for maintaining the structural integrity of the isolated EVs.

Additional descriptions of experimental techniques are provided in the Supporting Information file. The mass spectrometry proteomics data have been deposited to the ProteomeXchange Consortium via the PRIDE (Perez‐Riverol et al., 2022) partner repository with the dataset identifier PXD049702.

## 3 RESULTS

### 3.1 Method development and optimization

In the method development and optimization phase, we implemented orthogonal techniques, including TEM, NTA, and western blotting, in addition to LC–MS/MS‐based proteomic analysis for a comprehensive EV characterization and method efficiency evaluation (Figure 1).

#### 3.1.1 Optimization of resin‐to‐plasma ratio for balanced sample purification versus EV recovery

In both CC700‐based EV pre‐purification and Q‐Sepharose‐based EV fractionation steps, an investigation was performed to strike a balance between the purity of EV isolates (assessed using 1D‐polyacrylamide gel electrophoresis [1D‐PAGE] silver staining) and EV recovery (indicated by anti‐CD9 western blot) in the process of EV isolation. For CC700, we tested four resin‐to‐plasma (mg:µL) ratios: 2.5, 3.0, 3.5, and 4.0. The comparative analysis (Figure S1A) revealed that the 3.0‐mg/µL ratio provided a moderate efficiency level of free top abundance plasma protein depletion during the Capto Core‐based EV pre‐purification step, especially for albumin and IgG, while maintaining a strong CD9 signal. Since this is the initial step and larger EV amounts are favourable for subsequent fractionation, we selected the 3.0‐mg/µL ratio as an appropriate balance between the purity and recovery of EV isolates. Moving to the Q‐Sepharose SAX fractionation step, after initial tests, we selected two resin‐to‐plasma (mg:µL) ratios to evaluate and compare: 8.0 and 5.0. The acquired results (Figure S1B) demonstrated that the 8.0‐mg/µL ratio resulted in effective protein distribution across multiple fractions with strong CD9 signals present in the last two fractions, pH2 and FE, indicating a successful EV fractionation. On the contrary, at a ratio of 5.0 mg/µL, most of the proteins eluted in the FT fraction due to insufficient retention, and the EV recovery rate was much lower. We, therefore, selected a ratio of 8.0 mg/µL in the SAX fractionation step.

#### 3.1.2 Assessment of fractionation performance based on plasma protein distribution patterns

**FIGURE 2:**
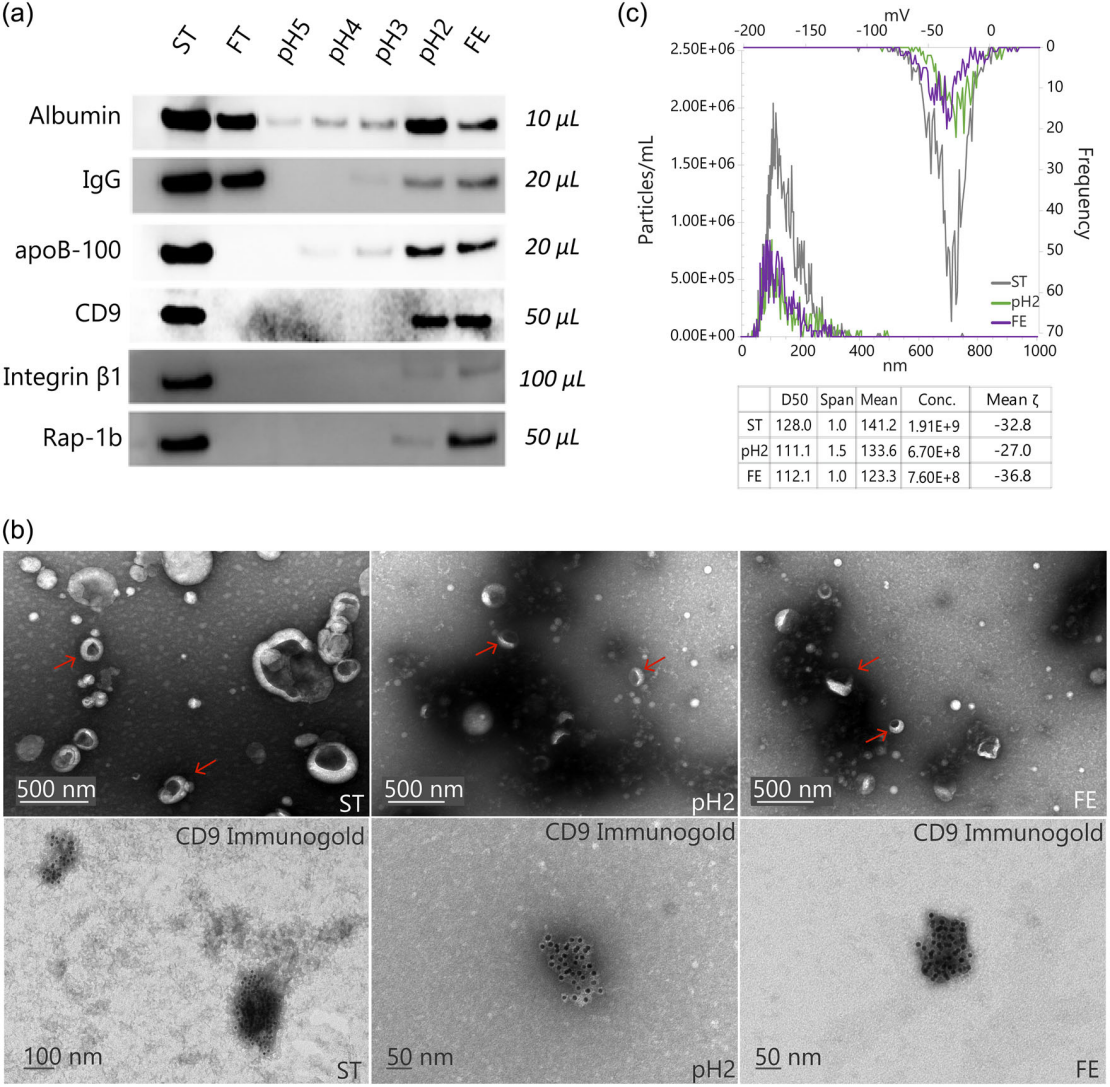
Orthogonal techniques used for EV characterization. (a) Western blot analysis demonstrates the fractionation/depletion patterns of six critical proteins, including three prevalent plasma proteins (albumin, IgG, and apoB‐100) and three EV‐related proteins (CD9, integrin β1, and Rap‐1b), across the ST and six discrete fractions. Italicized labels indicating sample volumes refer to the equivalent input plasma loaded per gel lane. (b) TEM and immunogold labelling: this panel illustrates TEM images of EV‐enriched samples (ST, pH2 and FE), highlighting the presence of characteristic EV‐like particles, predominantly displaying a ‘saucer’ or ‘cap’ shape with a dented centre. Immunogold labelling targeting the CD9 protein (lower row of images) further confirms the efficiency of EV isolation and fractionation. (c) NTA: This panel provides a profile of the size distribution, particle concentration, and zeta potential of EVs for the ST, pH2, and FE samples.

1D‐PAGE followed by silver staining (Figure S1B, ratio 8.0) and western blotting against three major plasma proteins, namely albumin, immunoglobulin G (IgG), and apolipoprotein B‐100 (apoB‐100) (Figure 2a) showed that the SAX‐based technique resulted in efficient fractionation of the major plasma proteins in the EV‐enriched pre‐purified ST sample. These patterns demonstrated the method's ability to efficiently separate EV sub‐populations and EVs from free plasma proteins and presumably other plasma components based on their charge. The method effectively enriches the residual top‐abundance‐free plasma proteins in specific fractions, thereby decreasing the dynamic range within EV‐rich fractions, an essential step for enhancing the depth, dynamic range, and quantitative accuracy of the downstream proteomic analysis. Contrary to aiming for the exhaustive depletion of predominant plasma proteins at the first step of EV enrichment using the multimode CC700 resin, this strategy seeks to establish a balance between (a) the fractionation efficiency in separating EVs from top abundance plasma proteins and separating EVs’ sub‐populations, and (b) EV recovery, which in turn, improves the performance of subsequent analytical readouts. Here are the observations made for several selected plasma proteins.

**Albumin** **–** Human serum albumin, which constitutes about 50%–60% of total plasma protein (Kratz & Elsadek, 2012), displays a unique elution pattern (Figure 2a). Upon analysing the 1D‐PAGE (∼66.7 kDa) and western blot bands, the highest observed intensity is present in the ST sample in both experiments, reflecting its status as the initial input. Similarly, both experiments showed that substantial amounts of albumin were detected in the FT and the pH2 fraction, while significantly lower signals were detected in the pH5 to pH3 fractions. Albumin has an isoelectric point (pI) of 4.5–5.0 (Phan et al., 2015), which makes the initial elution in FT (pH ∼7) reasonable per the ‘2 pH unit rule’ that is widely used in ion exchange chromatography (Dolan, 2017). The detection of an increased amount of albumin in the latter pH2 fraction might be indicative of albumin molecules that are bound to the surface of EVs, as proteins can exhibit altered binding behaviour based on their interaction with EV surface molecules or incorporation into EV lumen or EVP structure as their cargo (Baranyai et al., 2015; Buzás et al., 2018). Albumin also showed a moderate band intensity in the FE fraction. Distributing this most abundant plasma protein in several fractions narrows the dynamic range within each fraction, enhancing the potential for detecting less abundant proteins (e.g., EV‐related proteins).

**IgG** **–** Another major plasma protein, IgG, contributes to approximately 10%–20% of the total plasma protein (Kratz & Elsadek, 2012). Both the 1D‐PAGE (∼50 kDa, IgG heavy chain) and western blot experiments displayed a clearly observable band in the FT fraction, denoting that the majority of IgG was efficiently eluted at this early stage, which is due to its higher pI value of 6.5–9.5 (Chiodi et al., 1985). Interestingly, no bands were detected in the pH5 and pH4 fractions, but a faint signal in pH3 suggests the presence of a subset of IgG or IgG‐related complexes, and the band signal becomes more visible in the pH2 and FE fractions. The trace amounts of IgG in the latter fractions suggest likely interactions with EVs or their distinct post‐translational modifications, for example, glycosylation, or incorporation in the EV structure as cargo (Cobb, 2019). With IgG largely eluting in the FT fraction, the protocol ensures that its residual amount does not compromise the detection of less abundant proteins in the subsequent analyses.

**ApoB‐100**—Although apoB‐100 is less abundant in plasma than albumin or IgG, it is a major constituent of low‐density lipoproteins (LDL). Their close sizes to EVs usually cause ambiguities in distinguishing these two entities when using size‐based methods like SEC (Brennan et al., 2020). The apoB‐100 western blotting presents a weak signal in the pH4 fraction, indicating the starting point of its elution, which is followed by gradually intensified bands in the later fractions. Meanwhile, the protein abundance observed in these bands is substantially lower, compared to the ST sample. This obvious difference shows the method's effectiveness in fractionating LDLs. ApoB‐100's presence in late fractions with lower amounts might reflect potential interactions or co‐elution of lipoproteins with EVs.

To summarize, based on the provided observations, the developed approach efficiently fractionates plasma constituents, including free proteins, which in turn reduces the dynamic range to potentially enhance the characterization of EV sub‐populations. The enabled separation is particularly important for improving the depth of downstream proteomic analysis of EVs and EV sub‐populations isolated from relatively small plasma volumes (i.e., 50–200 µL).

#### 3.1.3 Validation of EV enrichment and fractionation based on semiquantitative assessment of selected proteins

To demonstrate the EV enrichment using the developed workflow, three notable EV‐associated proteins were investigated: CD9, integrin β1 and Rap‐1b. As per the MISEV (Minimal Information for Studies of Extracellular Vesicles) 2018 guidance (Théry et al., 2018), the chosen proteins are well‐qualified as EV‐associated markers due to their distinct roles and cellular localizations. CD9, a member of the tetraspanin superfamily, plays pivotal roles in EVs’ biogenesis, cargo selection, targeting, and uptake processes, commonly serving as an EV surface marker (Andreu & Yáñez‐Mó, 2014). Integrin β1, a membrane protein, is integral for cell adhesion and recognizes the extracellular matrix (ECM), making it a reliable indicator of the EVs’ membrane components. Rap‐1b, a cytosolic protein, is involved in intracellular signalling processes, further enhancing the diversity of proteins to represent various EV compartments. These three proteins, representing EV‐specific tetraspanin, membrane, and cytosolic components, respectively, provide an extensive validation spectrum for the presence of EVs.

**CD9** – Western blot results for CD9 reveal a distinct and strong band in the ST sample, indicating its significant abundance in the starting material (Figure 2a). Interestingly, post‐fractionation, only the pH2 and FE fractions manifest strong bands for CD9. This selective elution underscores that the majority of EVs are particularly enriched within these two fractions, thereby validating the efficacy of the fractionation procedure.

**Integrin β1** – The presence of integrin β1 in the pH2 and FE fractions underlines its potential as a reliable marker for the verification of EV presence, particularly when complemented by other established EV markers (Figure 2a). Its detection on the western blot shows a less intense signal compared to CD9 despite its plasma input volume being double that used for CD9. This difference may result from its relatively lower expression level in the parent cells or be ascribed to its more auxiliary role in EV biogenesis, as compared to CD9 (Toribio & Yáñez‐Mó, 2022).

**Rap‐1b –** Cytosolic protein Rap‐1b plays a crucial role in various cellular processes, including integrin activation and adhesion (Bromberger et al., 2019). Its detection in EV‐containing fractions underscores the intricate cargo‐sorting mechanisms inherent to EVs. As it is not a secreted protein, the detection of Rap‐1b in the EV‐containing fractions directly indicates its encapsulation within EVs, thus serving as evidence for efficient EV isolation and fractionation.

The notable variance in band intensity between the FE and pH2 fractions (Figures 2a and S2) suggests a potentially selective enrichment of EV sub‐populations with varied cargo content. This disparity may indicate the differential biogenesis pathways or cellular states from which these EVs originate. For instance, it is conceivable that EVs rich in Rap‐1b might be released during specific cellular events, such as cell‐to‐cell signalling or stress response, given Rap‐1b's involvement in cell adhesion processes. However, such hypothetical interpretations will need to be experimentally validated, which is beyond the scope of this study.

In conclusion, the western blot analysis effectively validates the presence and charge‐based fractionation of EVs and the three selected top abundance plasma proteins (see Figure S2 for the quantified optical densitometry analysis of the bands of interest). The distinct fractionation patterns observed for these proteins confirm the method's efficiency in separating plasma components, including free proteins and EVs, based on electrostatic interactions. Instead of seeking the total removal of the main plasma proteins, this approach aims to achieve a balance between the recovery of EV sub‐populations and the separation of EVs from the top plasma proteins, thus facilitating further analysis. The presented results of efficient charge‐based fractionation establish a solid foundation for the subsequent proteomic analysis.

#### 3.1.4 Characterization of EV morphology

TEM serves as a crucial technique for visualizing the morphology of EVs. The TEM images (Figures 2b and S3) of the seven sample types (i.e., ST, FT, pH2, pH3, pH4, pH5, and FE fractions) and a reference blank dPBS demonstrate the presence of characteristic EV‐like particles prominently in the ST sample, and pH2 and FE fractions. This observation, together with the western blot results shown in Figure 2a, further validates the effectiveness of the developed fractionation method.

Notably, these EV‐like particles display a characteristic ‘saucer’ or ‘cap’ shape with a dented centre. This unique morphology is a known property of EVs in TEM visualization (Zhao et al., 2021). The phenomenon is due to the drying step during the preparation phase, which partially collapses the vesicles, leading to the formation of saucer‐like structures.

The EV size spans from approximately 120 to 300 nm. Within the ST sample, certain instances of EV aggregation are evident, wherein multiple EV‐like particles appear to cluster together. Such aggregation tendencies are commonly reported (Rogers et al., 2020), often influenced by several factors, such as the proteins present on the EV surface and the specific conditions under which EVs are isolated or prepared.

Moreover, smaller, uniformly bright particles without dented centres are present in the EV‐containing samples. These particles, potentially representing co‐eluted lipoproteins or non‐vesicular extracellular particles (NVEPs) (Welsh et al., 2024), further align with our western blot observations, suggesting the co‐enrichment of specific plasma components.

To augment the specificity of our TEM imaging and further validate the presence of EVs, an immunogold labelling technique targeting the CD9 protein was employed. The resulting TEM images for the EV‐containing samples (ST, pH2, and FE) demonstrated the definitive binding of gold nanoparticles (10 nm) to the surface of the saucer‐shaped particles. The detected immunoaffinity‐based binding verified the presence of EVs and the efficacy of the chosen marker.

#### 3.1.5 Size distribution and zeta potential of EVs

NTA delivers a detailed assessment of particle size distribution, concentration, and surface charge attributes. Figure 2c presents a composite plot that includes an inset table, which concisely summarizes the key statistical measurements, elucidating the characteristics of particles in three EV‐enriched samples ST, pH2, and FE.

In the plot depicting particle size distribution versus concentration, originating from the lower left corner, the ST sample is observed to have a median size (D50) of 128.0 nm. Comparatively, the pH2 and FE fractions exhibit slightly smaller median sizes of 111.1 and 112.1 nm, respectively. The particle size distribution in the ST and FE samples shows a span value (a metric indicating the width of the size distribution) of 1.0, signifying an intermediate breadth in the size distribution curve. Conversely, the pH2 fraction shows a slightly wider distribution with a span value of 1.5 and a discernible tailing peak on the plot.

The count‐weighted mean sizes for the ST, pH2, and FE samples are measured to be 141.2, 133.6, and 123.3 nm, respectively. Additionally, the particle concentrations for these samples are recorded as 1.9E + 9, 6.7E + 8, and 7.6E + 8 particles/mL, respectively. This data indicates a nominal particle loss, possibly because of EV lysis caused by the harsh conditions in the pH2 and FE fraction before the buffer exchange process.

In the upper right section of the plot, which represents zeta potential against frequency, the ST sample exhibits an average zeta potential value of −32.8 mV. In comparison, the pH2 and FE samples record mean zeta potentials of −27.0 mV and −36.8 mV, respectively. The increased negative charge in the FE sample relative to the pH2 sample aligns with the expected outcomes based on the mechanism of anion exchange chromatography, i.e., the heightened charge densities of EVs in the FE fraction contribute to stronger binding to the SAX resin. It also indicates charge‐based EV sub‐populations, aligning with western blot findings.

It is worth noting that characterization techniques like NTA have limitations in detecting sub‐100‐nm particles, which may lead to discrepancies in size compared to previous reports on plasma EVs using other methods. While the Capto Core resin's average pore size is unlikely to significantly exclude smaller vesicles, partial depletion or aggregation effects cannot be fully ruled out. Future advancements in sub‐100‐nm EV characterization will further enhance these evaluations.

#### 3.1.6 Proteomic data analysis confirms method reproducibility and EV fractionation efficiency

**FIGURE 3:**
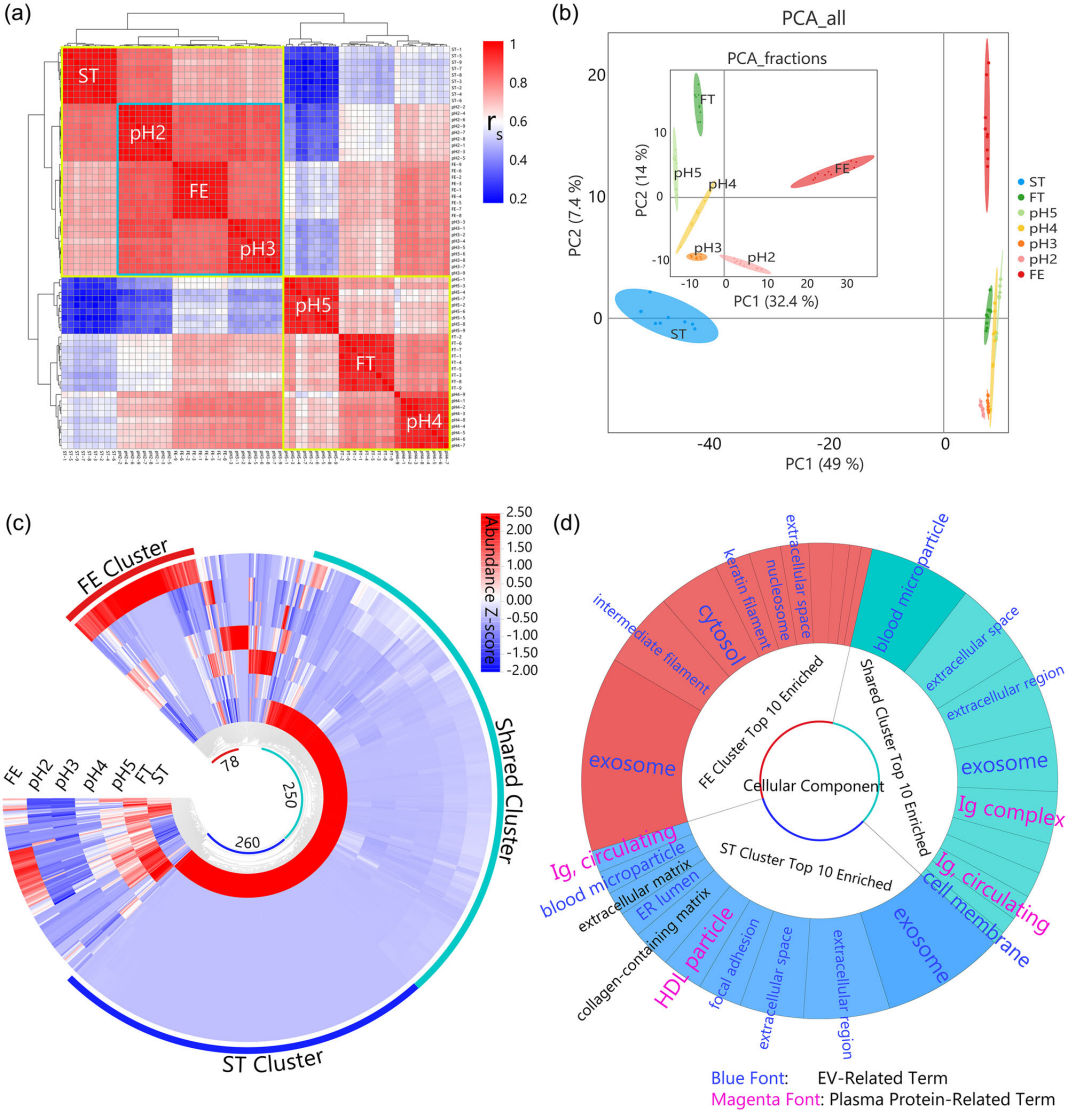
Multifaceted proteomic analysis for method development samples. (a) Inter‐correlation analysis highlights high reproducibility across sample datasets with Euclidean distance‐based unsupervised hierarchical clustering, revealing detailed grouping patterns among EV‐enriched fractions, for example, FE, pH2, and starting sample, ST. (b) Principal component analysis (PCA): The main figure visualizes distinct proteomic profiles of ST and all SAX fractions; the nested inset figure, excluding ST, emphasizes subtle proteomic profile differences between FE and pH2 and the method efficacy in fractionation. The ellipses represent 95% confidence levels. (c) Clustering heatmap demonstrates variations in protein abundances among samples, showcasing the fractionation method's ability to differentiate protein profiles and EV sub‐populations, highlighted by three distinct clusters: the FE cluster (red arc‐embraced sector), shared cluster (cyan arcs), and ST cluster (dark blue arcs). (d) The results of gene ontology (GO) enrichment analysis: the Krona pie chart in this section illustrates the 10 most significant GO terms for the three clusters identified in panel (c). Each GO term's sector area in the chart is proportional to its −log_10_(*p*‐value). The FE cluster is characterized by GO terms predominantly associated with EVs (indicated in blue font) coming from cytosol and organelle origins. In contrast, the shared cluster encompasses terms mainly related to blood‐cell‐originated (blood microparticle GO terms) and cell‐membrane‐origin vesicles, along with GO terms associated with plasma proteins, highlighted in magenta font. Notably, the ST cluster features terms related to high‐density lipoprotein (HDL) particles. This analysis demonstrates a higher rate of plasma protein depletion in the EV fractions compared to the ST sample. Furthermore, the distinct origins of EVs between the fractions suggest the capability of the developed technique to effectively fractionate EV sub‐populations.

Our advanced proteomic data analyses were crucial in characterizing the plasma‐derived EVs (Figure 3). The applied techniques included inter‐correlation analysis and principal component analysis (PCA), which not only demonstrated the reproducibility of our method but also suggested the relations among EV‐containing samples. Gene ontology (GO) enrichment analysis of the distinct protein clusters from the hierarchical clustering provided insights into the enrichment of varied EV‐related proteins and the depletion of plasma proteins within different EV‐containing samples, highlighting the efficacy of our fractionation approach in isolating specific EV sub‐populations. Moreover, the Venn diagram analysis was used to identify unique proteins in each EV fraction, shedding light on potential protein markers characteristic of distinct EV sub‐populations.

##### Inter‐correlation analysis demonstrates method reproducibility and sample grouping patterns

An inter‐correlation analysis using Spearman's rank correlation coefficient (*r_s_*) was performed on the label‐free quantification (LFQ) proteomic data to evaluate the method's reproducibility. Nine datasets derived from three preparation replicates and three LC–MS injection/analysis replicates for each sample type were subjected to the *r_s_* calculation, followed by a hierarchical clustering analysis. The heatmap generated (Figure 3a), particularly along the main diagonal, indicated high quantitative reproducibility within each sample's data set, thereby confirming the method's reliability.

The unsupervised hierarchical clustering analysis revealed clear grouping patterns of samples based on quantitative proteomic profiles (Figure 3a). The FE fraction initially demonstrated a close clustering and similarity with the pH3 fraction, and this combined FE–pH3 group subsequently clustered with the pH2 fraction. This trio was then grouped with the ST sample, finally forming a distinct dominant cluster. This FE–pH3–pH2–ST cluster was markedly separated from the other predominant cluster, which consisted of pH4–FT–pH5.

The arrangement within the hierarchical clustering suggests both similarities and differences in the proteomic profiles. Notably, the FE fraction did not directly cluster with pH2, implying the potential presence of distinct EV sub‐populations. However, the formation of a dominant cluster encompassing ST, pH2, pH3, and FE suggests potential EV‐like molecular features in the pH3 fraction, even though the specific molecular profiles have not been confirmed by other techniques, which might be influenced by the method sensitivity and EV marker specificity. This observation proposes a subtle proteomic similarity and diversity among these fractions, providing a rationale to explore the pH3 fractions further in the pilot profiling experiments of clinical samples. The observed clustering not only underscores the uniqueness of the molecular composition in each fraction but also suggests a collective proteomic phenotype that could be instrumental in differentiating EV‐rich fractions from other fractions with enriched high abundance free plasma protein species.

##### Principal component analysis reveals proteomic variability among samples

PCA provides a holistic visualization of the proteomic profiles across samples. From the main ‘PCA_all’ panel (Figure 3b), the ST sample occupies a unique position along the PC1 dimension, which accounts for 49% of the total variance. This delineation underscores the distinct proteomic composition of ST when compared with the SAX fractions.

To further explore the fractions, we removed the ST sample for separate analysis. The results, as illustrated in the nested ‘PCA_fractions’ panel, reveal a complex landscape. The FE fraction is located on the far right of the PC1 axis, which takes up 32.4% of the total variance, whereas the pH2 fraction appears around the midpoint. Their positions suggest distinct proteomic compositions and abundances, potentially correlating to different EV sub‐populations.

The variance observed along the PC2 axis of the inset plot, representing 14% of the total variance, further distinguishes the proteomic profiles of the pH2 and FE fractions. This axis highlights differences in addition to those captured by PC1, especially emphasizing the differential nature of these two EV‐enriched fractions.

The elongated ellipses show 95% confidence for each sample. Notably, all nine data points corresponding to replicate experiments for a given sample group fall within their respective confidence ellipse, which is indicative of the reproducibility of the method. The clear separation between samples, particularly given the lack of overlap between ellipses, reflects their distinct variance patterns, reinforcing the efficacy of our fractionation method.

By linearly reducing the dimensionality of the original protein abundance data matrix, PCA allowed us to distil the multivariate data into principal components. This process reveals sample variability patterns that were not immediately discernible from the original proteomics data, facilitating a more straightforward interpretation of complex biological systems.

##### Unsupervised hierarchical clustering unveils distinct and most abundant protein groups

Utilizing unsupervised hierarchical clustering on the LFQ proteomic data demonstrated significant differences in protein abundances among the samples, which confirms the fractionation efficiency of the developed method. This analysis reveals distinct protein clusters characteristic of different samples (Figure 3c). Two major clusters demonstrated by their highest abundance of proteins were identified. The first cluster encompasses 78 proteins most abundant in the FE fraction, labelled as ‘FE cluster’ bracketed by a pair of outer and inner red arcs (Figure 3c). The increased number of IDs could be due to the reduced dynamic range of the FE sample and, therefore, a lower extent of ionization suppression than in the analysis of the ST sample, which in turn resulted in the increased profiling depth for the fractionated EV sample. The second, a considerably larger cluster, was predominantly abundant in the ST sample. However, a more detailed cross‐sample (i.e., radial) inspection revealed that this vast cluster could be further dissected into two sub‐clusters. The first of these sub‐clusters displayed a moderate yet noticeable abundance in the FE and pH2 fractions, leading us to term it the ‘shared cluster’ (cyan arcs), which contains 250 proteins. Conversely, the second sub‐cluster of 260 proteins, labelled as ‘ST cluster’ (blue arcs), was exclusive to the ST sample. These low‐abundance proteins were not detected in the fractionated samples, possibly due to smearing across several fractions, which made their signal below the detection limit. We performed a GO enrichment analysis focusing on cellular component terms to gain deeper insights into the roles and potential significance of the proteins within these clusters.

##### Gene ontology enrichment analysis reveals cellular components of proteins enriched in the clusters

The Krona pie chart (Figure 3d) integrated the results of the individual GO enrichment analysis, providing visualization of the top 10 most significantly enriched cellular component terms for previously identified FE, shared, and ST clusters. Each term's area reflects its statistical significance as evaluated by the negative base 10 logarithm of the *p*‐value.

**ST cluster –** The ST cluster prominently features the term ‘exosome’ as the largest sector, indicating efficient EV enrichment. However, alongside this dominating EV‐associated term and several other EV‐related broader GO terms (i.e., ‘blood microparticles’, ‘extracellular space’, ‘extracellular region’), we also identified terms indicative of secreted plasma proteins, including ‘high‐density lipoprotein (HDL) particle’ and ‘circulating immunoglobulin complex (Ig, circulating)’. This combination of GO terms reflects the higher complexity level of the ST sample before it was subjected to the subsequent SAX fractionation.

**Shared cluster –** In the shared cluster, the most significant term is ‘blood microparticle’, which was closely followed by the terms ‘extracellular space’, ‘extracellular region’, and ‘exosome’. EVs predominantly originated from blood cells, and endothelial cells were likely responsible for the presence of proteins that were allocated to this cluster. Other GO terms in this cluster, such as the fourth‐placed ‘exosome’ and the tenth‐placed ‘cell membrane’, prove the efficient EV enrichment in both the EV fractions and the unfractionated ST sample. The prominent sectors corresponding to the terms ‘immunoglobulin complex (Ig complex)’ and ‘Ig, circulating’ indicate the presence of antibodies in the studied samples. These results could be attributed to EVs' role as intercellular communicators, where they might harbour immunoglobulins, either as cargo or associated with their membranes (possibly due to the high abundance of immunoglobulins in blood and the so‐called ‘sponge effect’ (O'Brien et al., 2020)), implicating possible immune interactions or modulations.

**FE cluster** – Within the FE cluster, the term ‘exosome’ once again dominates. However, the rest of the terms are more diverse. The presence of the ‘intermediate filament’ underscores the cytoskeletal dynamics within EVs, perhaps hinting at their role in maintaining vesicular structure or at the specific biogenesis pathways. The terms ‘cytosol’, ‘nucleosome’, ‘proteasome core complex’, and other organelle‐related terms further highlight the rich internal content of the EVs isolated in this fraction, emphasizing their role as carriers of diverse cargos (see the full version of Krona pie chart in Figure S4). The identification of terms like ‘nucleosome’ and ‘proteasome’ may suggest specific biological processes, such as apoptosis, as the possible origin of these EVs. However, further studies are needed to clarify their exact origins.

In conclusion, the GO term enrichment analysis confirms the efficient EV enrichment and fractionation by the developed method. While demonstrating its predominant EV association, each cluster also offers additional insights into the diverse molecular composition of EVs and their sub‐populations, which has the potential to augment our understanding of EV biology.

##### Distinct proteomic profiles of EV fractions

**FIGURE 4:**
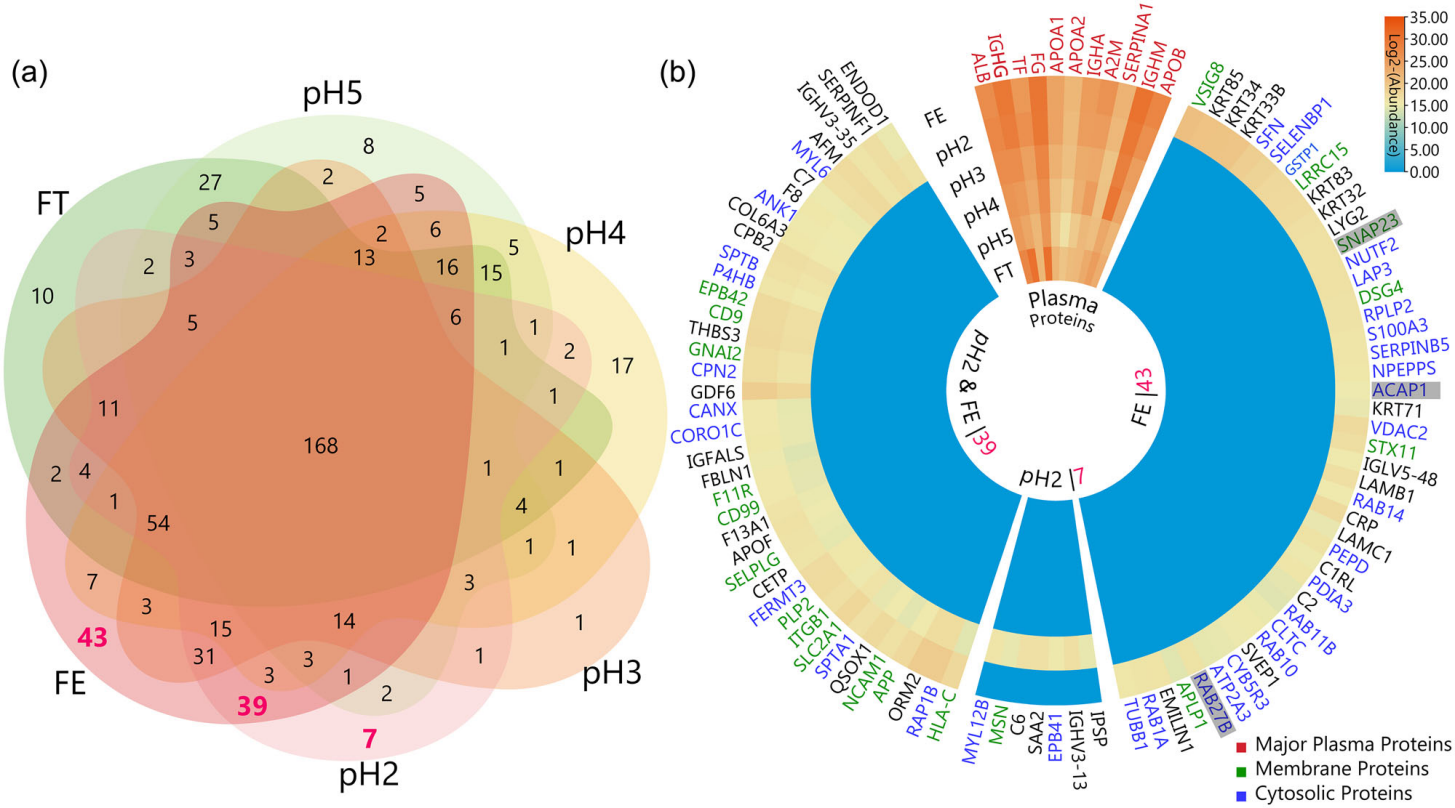
Detailed proteomic profiling and sub‐cellular origin annotation of unique proteins from EV‐enriched fractions. (a) Venn diagram illustrates the unique and commonly detected proteins between the examined fractions. For example, 43 and seven unique proteins were identified in the FE and pH2 fractions, respectively, highlighting the distinct proteomic features of these EV‐rich fractions. Thirty‐nine proteins were commonly detected in both the FE and pH2 fractions. (b) Circular heatmap displays the relative abundance levels of these pinpointed proteins. Proteins from cell membranes and cytosol, indicative of EV constituents, are shown in green and blue fonts, respectively. Eleven major plasma proteins (red font) are also mapped alongside, providing insights into their elution patterns.

To further dissect the proteomic data, we conducted a detailed mapping of 11 plasma proteins and 89 uniquely detected proteins in EV fractions (Figure 4). We first plotted a Venn diagram (Figure 4a) for all fractions to extract those proteins that are uniquely present in pH2 and FE or shared by the two fractions. As a result, 43 and seven proteins were uniquely identified in the FE and pH2 fractions, respectively. This uniqueness could be attributed to the reduced dynamic range of the fractionated sample, allowing for a deeper proteomic exploration. Meanwhile, 39 proteins were commonly detected in FE and pH2 fractions. The relative abundance of these 89 proteins in total was subsequently illustrated in a circular heatmap (Figure 4b).

We also mapped the distribution of 11 plasma proteins, including the most abundant albumin and IgG (Kratz & Elsadek, 2012), as well as apolipoproteins, in the same heatmap. The precise analysis of these proteins provided a clear perspective on their elution patterns in SAX‐based fractionation. Albumin (*ALB*) and IgG (*IGHG*) were detected across all fractions at various levels, and their abundance distributions, along with apoB‐100 (*APOB*), correlate well with our western blot results (Figure 2a), reinforcing the consistency between the two orthogonal analytical techniques and emphasizing the reliability of our proteomic findings.

Next, we explored the subcellular origins of the 89 unique proteins. Those originating from cell membranes or the cytosol, likely to be EV constituents, are highlighted in green and blue, respectively, to indicate their potential relevance to EVs. Within these colour‐coded groups, several proteins with shadowed fonts in the heatmap and discussed below stood out due to their association with intracellular membrane transport systems. These proteins are particularly involved in pathways such as endosomal maturation, which are reportedly linked to the formation of EVs, particularly influencing their biogenesis, intracellular trafficking, and the specific sorting mechanism of cargos (Gandham et al., 2020). Here are a few examples of such proteins.

*SNAP23* is a member of the SNARE (soluble N‐ethylmaleimide‐sensitive factor attachment protein receptors) protein family and plays a critical role in vesicle‐membrane fusion processes. While its most established function is in mediating vesicle trafficking in non‐neuronal cells, its involvement in the fusion of multivesicular bodies (MVBs) with the plasma membrane was recently reported (Qian et al., 2021). This fusion is an essential step in the secretion of exosomes, which are a subtype of EVs. Thus, *SNAP23* may serve as a key player in ensuring the efficient release of exosomes into the extracellular environment. *ACAP1* is an ArfGAP protein, which means it is a GTPase‐activating protein for ADP‐ribosylation factor (Arf) proteins. Arf proteins are small GTPases that play vital roles in vesicular trafficking. *ACAP1* has been shown to regulate endocytic recycling by acting on Arf6, which is involved in endosome dynamics and the recycling of endocytic vesicles back to the plasma membrane (Li et al., 2007). Given that the endocytic pathway is reportedly linked to the formation of MVBs (Piper & Katzmann, 2007) and the subsequent release of exosomes, *ACAP1*’s involvement in regulating this pathway could imply a role in EV biogenesis. *RAB27B* is a member of the Rab family of small GTPases. Known as a late endosomal protein, it has a well‐established role in the secretion of exosomes. By regulating the docking and fusion of MVBs to the plasma membrane, *RAB27B* is directly involved in the final steps of exosome secretion (Gurung et al., 2021). Other RAB proteins in the heatmap, including *RAB10*, *RAB11B*, and *RAB14*, have also been linked to vesicular trafficking and may influence EV biogenesis by governing the movement and fusion of vesicles.

Understanding the roles of the above‐discussed proteins in EV biogenesis and functions can provide insights into the complex and diverse molecular mechanisms that govern vesicle formation, trafficking, release, cell–cell communication, and involvement in pathologies.

In conclusion, the development and optimization of the presented method allowed us to enhance the analysis of plasma‐derived EVs, enabling more detailed investigations of the protein content of EVs and EV sub‐populations. These improvements were achieved through the development of simplistic but efficient chromatography‐based isolation and fractionation techniques, with the performance validated using orthogonal methods, such as 1D‐PAGE, western blotting, and TEM. The effectiveness of our EV fractionation method is demonstrated by the enrichment of CD9‐specific EVs, which was assessed through the recovery of EV markers (i.e. CD9), reduction of the amount of free plasma proteins in EV isolates, and improved identification of unique proteins in the conducted proteomic analysis. These combined performance assessment approaches demonstrated the reliability of our data, offering a solid framework for follow‐up studies of EV proteomes.

### 3.2 Evaluation of method applicability to the analysis of clinical samples

Next, we advanced our experiments from method development and performance assessment to the pilot method evaluation using a small set of clinical samples. We implemented the developed method to obtain EV fractions from a cohort of clinical samples, comprising 10 PCa patients and 10 healthy age‐matched controls. Utilizing the optimized nanoLC–MS/MS and data processing methods, we acquired LFQ data from the EV‐enriched fractions, notably pH2 and FE, and also from the pH3 fraction, given its proximity to the pH2 fraction and its potential to isolate specific EV sub‐populations. The total number of quantified proteins in each of these fractions for both PCa patient and control groups is available in Table S4. Further data analyses were divided into two directions. First, we consolidated the LFQ data from these fractions and then conducted a differential protein abundance analysis using the combined quantitative proteomic data. The goal was to determine DAPs that might serve as distinct signatures that may be potentially indicative of PCa versus controls. Meanwhile, another initial goal was to use the EV sub‐populations for a more precise discovery of proteomic alterations relevant to the disease. Therefore, we also conducted differential analysis using the quantitative proteomic data for each individual fraction across all analysed samples.

#### 3.2.1 Identification of differentially abundant proteins (DAPs) between PCa versus control samples

##### Data evaluation for selecting an appropriate differential analysis algorithm

To initiate the pilot studies of a small cohort of clinical samples, we examined the combined LFQ dataset for the extent of data missingness across the acquired datasets (Figure S5A). With an average missing percentage of 18.3% sample‐wise, this value is typical for proteome‐scale datasets, especially for those derived from data‐dependent acquisition‐based LFQ analysis, which we also found reasonable for the conducted experiments.

To maintain the integrity and reliability of the analysis, based on initial manual assessment, we selected the following threshold for quantitated protein features across all investigated samples (i.e., 10 PCa patients = the Disease group and 10 healthy controls = the Control group): proteins that exhibited more than seven missing values within each group were filtered out. This approach ensured that each protein had a minimum of three valid values within each group, which allows for more reliable intra‐group variance calculation for the differential analysis. While not universally adopted, using at least three valid values in exploratory proteomics studies is a pragmatic approach, particularly when dealing with small sample sizes or low‐abundance proteins. This method allowed us to detect molecular alternation patterns that might be biologically relevant but are not consistently observed across all samples due to technical or biological variability. Given the paired nature of our samples (age‐matched disease and control donors), we took a conservative approach by avoiding imputation of missing values. Imputation can inadvertently introduce artificial uniformity, potentially overshadowing actual differences in protein abundance that are critical in paired analysis.

Diving deeper into protein feature‐wise attributes, we employed the Shapiro–Wilk test (Yap & Sim, 2011) to gauge the data distribution normality within each group, coupled with Levene's test (Gastwirth et al., 2009) to assess homoscedasticity (i.e., consistency of variances) between them. These tests are foundational, as the choice of differential analysis tools depends on data distribution. Applying an adjusted *p*‐value threshold of 0.05, the heatmap from these tests (Figure S5B) demonstrated that, for the majority of proteins, our log_2_‐transformed data maintained satisfactory levels of homoscedasticity. However, in terms of normality, only approximately half of the proteins conformed to the criterion. The remaining proteins exhibited distributions that were close to, but not fully consistent with, a normal distribution.

Given the data's characteristics, we selected the LIMMA package in R to conduct differential expression analysis. LIMMA, with its linear modelling foundation and empirical Bayes (EB) methodology, is particularly capable of efficiently processing complex datasets like ours. One notable strength of LIMMA is its robustness against deviations from normality. While traditional differential analysis might be heavily influenced by such deviations, LIMMA's EB approach mitigates this issue by borrowing strength across all features, providing more stable estimates (Smyth, 2004). In our case, the EB method leverages information from the entire set of proteins, facilitating a more precise variability estimation. Consequently, even though our data is not perfectly normally distributed and there is only a moderate to small sample size, LIMMA can reliably discern differential expressions, making it an optimal choice for our dataset (Ritchie et al., 2015).

##### Similarities and differences in EV proteomic profiles between disease and control samples

**FIGURE 5:**
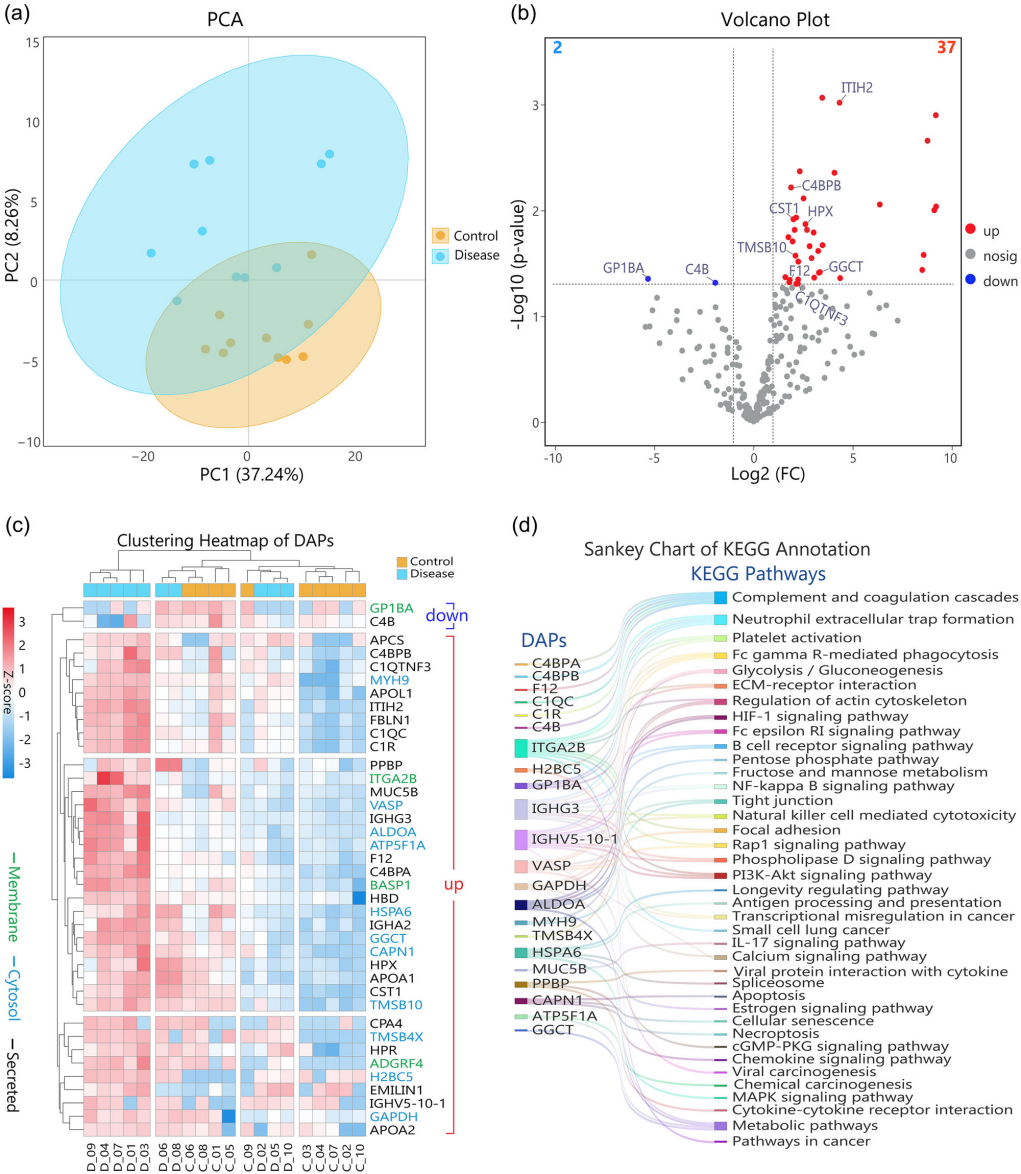
Comparative proteomic analysis of disease versus control groups. (a) PCA plot illustrates the variance in the full proteomic profiles between disease and used here control groups. The first two principal components, PC1 and PC2, represent 37.2 and 8.3% of the total variance. The size of the 95% confidence ellipse indicates variability within each group, with the disease cohort showing a broader range in proteomic expression. (b) Volcano plot displaying differentially abundant proteins (DAPs) in prostate cancer, highlighting 37 upregulated and two downregulated proteins with a fold change >2 or <0.5 and a *p*‐value < 0.05 criteria. (c) Hierarchical clustering heatmap: Row‐wise analysis reveals distinct clusters based on protein abundance, including a separation of the two downregulated proteins from the rest of the upregulated protein clusters. The heatmap also displays sub‐clusters of secreted, cytosolic and membrane proteins, suggesting their varied roles in the disease and relations to EVs. Column‐wise analysis illustrates distinct clusters among the disease and control samples, reflecting the complex interplay of factors influencing protein expression in these groups. (d) KEGG pathway annotations reveal cancer‐relevant pathways for 22 out of 39 DAPs.

A PCA analysis was performed to visualize the multivariate data, to allow us to discern potential patterns that may justify further investigation. The PCA plot (Figure 5a) indicates that the first principal component (PC1) accounted for 37.24% of the total variance, while the second principal component (PC2) explained an additional 8.26%. The ellipses on the PCA plot, corresponding to the 95% confidence intervals of the data points, indicate the variability within each group. For the Disease group, the ellipse is notably larger, which represents a higher variance in a diverse proteomic profile among patients. In contrast, the Control group displays a smaller ellipse, reflecting a more uniform distribution of quantitative proteomic profiles. Meanwhile, the overlapping region of the ellipses suggests a subset of proteins that are similar in their abundance in both groups. Taken together, these factors indicate the sophisticated mechanisms that shape the proteomes in both groups. The interplay between disease heterogeneity, individual biological variability, or even medical conditions highlights the great challenge of delineating disease states unequivocally. This complex interplay of shared and distinct proteomic patterns necessitates a deeper exploration of the biomolecular underpinnings of the disease.

##### Differential proteomic analysis of EVs derived from PCa and control groups

Next, we conducted differential abundance analysis to pinpoint specific proteins, that is, DAPs, which might play key roles in PCa manifestation, especially through the detection in EV populations. Then, we developed the volcano plot (Figure 5b). This graphical representation compares the magnitude of change in protein abundance (log_2_ fold change) with the statistical significance of the difference (*p*‐value). A *p*‐value of 0.05 and |log_2_FC| > 1 threshold was set for filtering. As a result, 37 upregulated and two downregulated proteins were identified as DAPs. Notably, several of these DAPs, including the ones that we briefly discuss below, have previously been associated with PCa progression, underscoring the validity and potential impact of our findings.

A recent study suggests that *ALDOA* (fructose‐bisphosphate aldolase A) plays a significant role in the progression of PCa. The research indicates that the *ALDOA* metabolism pathway could be a potential target for regulating PCa proliferation (Kuang et al., 2021). This study provides insights into the importance of *ALDOA* in PCa's metabolic processes, suggesting its potential as a therapeutic target. Another study concludes that the coding variation in *CPA4* (carboxypeptidase A4) may confer an increased risk of intermediate‐to‐high‐risk PCa among younger patients. This research indicated that certain genetic variations within *CPA4* are associated with a more significant likelihood of developing more aggressive forms of PCa, particularly in younger individuals (Ross et al., 2009). Another study suggests that EGF receptor‐mediated prostate tumour progression is dependent on *STAT3*, which regulates the expression of *VASP* (motility‐limiting vasodilator‐stimulated phosphoprotein), and the apoptosis nexus *CASP3*. This indicates that *VASP* may play a role in the migration and invasion processes of prostate carcinoma cells, contributing to the disease progression (Zhou et al., 2006). Another relevant study suggests that in a PCa xenograft model, the loss of hormone dependence is marked by irreversible histological alterations associated with an expression of secretory *MUC5B* (mucin‐5B) and other secretory mucins, *MUC2* and *MUC6*, independent of the histological differentiation sub‐type (Legrier et al., 2004). This indicates that *MUC5B* may play a role in the progression of hormone‐refractory PCa. Other proteins, including *GAPDH* (glyceraldehyde‐3‐phosphate dehydrogenase), also show significant functions in cancer biology. One recent study showed that *GAPDH* is commonly upregulated in various types of cancer and potentially required for cancer cell growth and tumour formation (Guo et al., 2013). Additionally, while certain proteins did not emerge as DAPs based on our threshold, they hovered close to the threshold limit, suggesting their potential relevance. Among them, one protein, *YWHAZ* (14‐3‐3 protein zeta/delta), deserves special mention. A recent study used integrative tissue‐omics to identify high *YWHAZ* expression subgroups, correlating with a high risk of disease progression, making *YWHAZ* a molecular biomarker associated with aggressive PCa (Lage‐Vickers et al., 2021).

In conclusion, our proteomic analysis of liquid‐biopsy‐based EV isolates provides valuable insights into the protein landscape of PCa. The identification of DAPs, coupled with the validation from existing literature, underscores the robustness of our findings. Our results offer a foundation for future studies to delve deeper into the mechanistic roles of these proteins, explore their potential as therapeutic targets, and contribute to our understanding of PCa progression.

##### Hierarchical clustering analysis of DAPs demonstrate intricate correlations of samples

Unsupervised hierarchical clustering analysis of the identified DAPs offers insights into the molecular alterations, which can be potentially associated with the disease (Figure 5c). The row‐wise analysis (protein‐centric) of the generated heatmap revealed a distinct clustering pattern, reflecting the complex regulation of protein expression in response to disease, at least to some extent based on this proof‐of‐concept studies. A smaller cluster of downregulated proteins *GP1BA* (platelet glycoprotein Ib alpha chain) and *C4B* (complement receptor type 1) was clearly separated from a larger cluster of upregulated proteins, highlighting a significant difference in protein abundance between control and disease conditions.

Within the upregulated proteins, the heatmap further revealed three distinct subclusters, each representing unique protein groups with potential implications in the disease mechanism. The upper one was predominantly composed of extracellular secreted proteins (black fonts), suggesting a role for these proteins in extracellular signalling and interactions that are possibly linked to the disease's pathophysiology.

The middle cluster, enriched with cytosolic proteins (blue fonts), including *VASP, ALDOA, ATP5F1A, HSPA6, GGCT, CAPN1*, and *TMSB10*, as well as two membrane proteins (green fonts) *ITGA2B* and *BASP1*, indicates alterations in cellular processes or pathways. These proteins, together with those in the lower cluster with cytosolic *TMSB4X, H2BC5, GAPDH*, and the membrane protein *ADGRF4*, are indicative of their origin from EVs, adding a layer of our understanding of EV's involvement in the disease.

On the other hand, the column‐wise clustering (sample‐centric) demonstrates a complex interplay of factors influencing protein abundance levels that do not completely separate the Disease and Control groups. Notably, the first cluster is comprised exclusively of five disease samples, which share the highest expression levels of these DAPs, suggesting a potential subset of the patient‐derived samples characterized by a unique protein expression signature. The second cluster represents a mix of two disease and four control samples, with the second highest level of DAPs’ abundance, indicating that specific control samples might share expression patterns with the disease subset. The third cluster includes a singular control sample C_09, along with three disease samples, occupying an intermediate position in terms of DAP expression levels. Finally, the fourth cluster is entirely composed of five control samples, which exhibit the lowest expression of the DAPs, possibly delineating a protein expression profile characteristic of the absence of disease.

These results suggest that while there is a differential expression of proteins associated with the disease, there is also considerable overlap between control and disease samples in the acquired data, potentially due to the heterogeneity of the disease, individual biological variability, variability in the disease progression and manifestation and possibly insufficient depth of proteomic profiling based on the used here sample amount and current LC‐MS technologies. The presence of disease‐only and control‐only clusters may reflect specific pathophysiological states or sub‐types within the broader disease category. In contrast, the mixed clusters could indicate that the disease process is not uniform across all patients. Additionally, the absence of age‐related patterns within the clusters (which we also assessed) may imply that age is not the primary factor driving the proteomic differences observed, or it may be overshadowed by the disease's impact or the biological variability among individuals. However, the number of samples used in this pilot study was limited, which does not allow us to draw significant conclusions based on age or disease status. Future studies should consider these factors, incorporating larger sample sizes and stratification by disease stages and subtypes, to gain a clearer understanding of the molecular landscape shaped by the disease.

##### Mapping the results of differential proteomic profiling to transcriptomic TCGA data

To add a level of verification to our proteomic analysis and enhance our comprehension of PCa's molecular dynamics manifested in EVs and EV sub‐populations, we examined DAPs identified in our study against the publicly available transcriptome data from TCGA (Cancer Genome Atlas Research Network et al., 2013). Through the UALCAN portal (Chandrashekar et al., 2017; Chandrashekar et al., 2022), we accessed TCGA's repository of PCa to investigate the transcriptional profiles corresponding to these DAPs.

A subset of these proteins, including *C4BPB*, *GGCT*, *F12*, *ITIH2*, *CST1*, *HPX*, *TMSB10*, and *C1QTNF3*, demonstrated significantly upregulated transcriptional patterns in the TCGA dataset (Figure S6). The correlation between our proteomic findings and the TCGA transcriptional data provides an additional layer of validation for the eight DAPs. This correspondence suggests that these proteins play a notable role in PCa and could be further investigated as potential biomarkers or therapeutic targets. The alignment of our results with TCGA data underscores the potential relevance of these proteins in the context of PCa. It also suggests that the combined multi‐omic analysis of proteomic and genomic data can be a powerful approach in the ongoing effort to understand the complexity of cancer biology and to develop more effective cancer diagnostic techniques and treatments.

In addition, we plotted receiver operating characteristic (ROC) curves of these eight selected DAPs for their differential abundance as potential diagnostic biomarker candidates upon appropriate additional validation (Figure S7A). Interestingly, *ITIH2* shows excellent diagnostic accuracy with an area under curve value (AUC) of 0.91. *C4BPB* and *CST1* demonstrate promising performance, with AUCs of 0.82 and 0.80, respectively. *GGCT*, *C1QTNF3*, *C1R*, and *HPX* display fair to good efficacy, with AUCs between 0.75 and 0.79. *F12*, with an AUC of 0.68, shows limited discriminative power, highlighting its constrained utility as a standalone biomarker. In addition, a ROC curve for a signature protein set from those with AUC greater than 0.80 (i.e., *ITIH2* and *C4BPB*) shows a combined AUC of 0.91 (Figure S7B). These proteins may be considered for future validation and careful monitoring in potential follow‐up studies using larger sample cohorts.

##### KEGG (Kyoto Encyclopaedia of Genes and Genomes) pathway annotation of DAPs in cancer biology

Proteins serve as critical mediators of cellular function and are central to the processes that govern cancer progression and tumorigenesis. Examining the pathways in which these proteins are involved provides a broad view of the roles that these proteins may play within the complex network of oncogenic pathways (Figure 5d). This approach is instrumental in elucidating the multifaceted interactions and processes that underlie cancer development and progression. Below, we discuss in detail *C4BPB*, *F12*, and *GGCT* DAPs and the related protein–protein interaction pathways and their relevance to PCa biology based on reported publicly available TCGA and KEGG data.

*C4BPB* (C4b‐binding protein beta chain) and *F12* (coagulation factor XII) proteins both play roles in the complement and coagulation cascade pathway of the immune system. These proteins, crucial to immune response and inflammation, are highly relevant to tumour biology. The immune system, particularly the complement system, is a double‐edged sword: while it is essential for neutralizing foreign threats, its anomalies can inadvertently aid tumour growth. This ambiguous role of the complement system in oncology has been reported (Ricklin et al., 2010). Recent studies explored the polyphosphate‐Factor XII (*F12*) pathway in the specific context of PCa‐associated thrombosis. The research revealed that this pathway is an active participant in cancer progression. In PCa, the polyphosphate‐Factor XII was identified as a key driver of thrombotic events (Hisada et al., 2015; Nickel et al., 2015). This association emphasizes the pathway's potential as a therapeutic target, offering avenues to mitigate thrombosis risk while addressing tumour growth and spreading.

*GGCT* (gamma‐glutamylcyclotransferase), a key enzyme in the gamma‐glutamyl cycle, is significant in cancer metabolism and therapy resistance. Identified within KEGG's metabolic pathways, its role extends beyond maintaining cellular redox balance, directly influencing tumour drug resistance (Traverso et al., 2013). Further, based on the *GGCT*’s direct contribution to tumour proliferation, inhibiting *GGCT*, particularly in *GGCT*‐overexpressing tumours, including PCa, presents a promising therapeutic strategy (Ii et al., 2018). This dual functionality of *GGCT*, from metabolic regulation to influencing tumour growth and therapy response, highlights its potential as a therapeutic target. Its enzymatic activity, especially in the context of overexpression, offers a unique vulnerability in cancer cells, proposing a potential shift in treatment approaches.

Building upon these insights into the DAPs mapped to KEGG pathways, we found it important to explore other proteins that, though not directly associated with established pathways, display significant relevance in cancer progression based on the reported TCGA data. The observed overexpression of *ITIH2* (inter‐alpha‐trypsin inhibitor heavy chain H2) in PCa underscores a complex interplay of molecular and cellular mechanisms. *ITIH2*, known for its involvement in stabilizing the ECM, may reflect critical adaptations within the tumour microenvironment, potentially facilitating structural changes conducive to tumour growth and metastasis (Hamm et al., 2008). Intriguingly, the correlation between *ITIH2* and oestrogen receptor expression opens avenues for deeper exploration, particularly in PCa, where hormonal dynamics are pivotal (Bader et al., 2018). Given the role of oestrogens in men, often altered significantly in PCa either due to the disease's hormonal manipulations (such as androgen deprivation therapy) or the tumour's local synthesis of oestrogens via aromatase, *ITIH2* overexpression might be an unforeseen consequence of these hormonal shifts (Ellem et al., 2004; Mostaghel et al., 2007).

#### 3.2.2 Differential proteomic analysis of individual fractions

**FIGURE 6:**
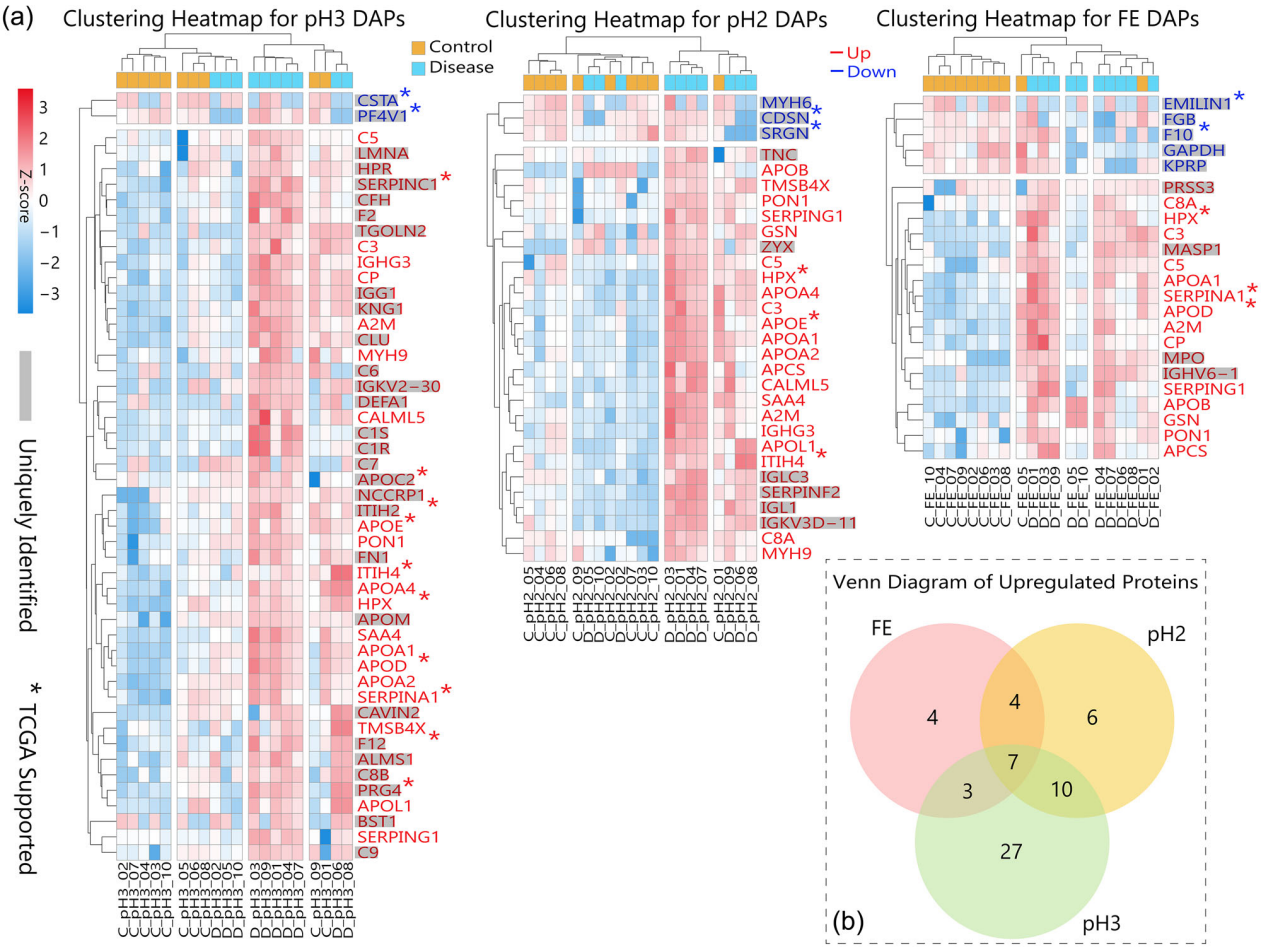
Hierarchical clustering and comparison of DAPs identified in the three EV‐rich fractions. (a) Hierarchical clustering heatmaps of DAPs identified in the pH3, pH2, and FE fractions, respectively. Uniquely identified proteins are shown in shadowed font. Asterisks indicate the proteins represented by the corresponding transcriptomic profiles in the TCGA data available for PCa. (b) Venn diagram of the upregulated proteins for the three EV‐rich fractions.

Applying uniform data analysis procedures and criteria across the three individual EV‐rich fractions resulted in the determination of differentially represented proteins in PCa and control samples. The number of DAPs identified were as follows: FE (18 upregulated and five downregulated), pH2 (27 upregulated and three downregulated), and pH3 (47 upregulated and two downregulated) (Figure 6a). Notably, all downregulated proteins were uniquely identified within their respective fractions. Among these, *CSTA* and *PF4V* in the pH3 fraction, *CDSN* and *SRGN* in the pH2 fraction, and *EMILIN1* and *F10* in the FE fraction exhibited expression patterns consistent with significant differences observed in their corresponding TCGA transcriptome data.

In the analyses of upregulated proteins, a Venn diagram was used to elucidate the unique and overlapping DAPs across the three fractions (Figure 6b). This analysis revealed seven proteins, *A2M*, *C3*, *PON1*, *APOA1*, *C5*, *SERPING1*, and *HPX*, as common across all fractions, with *HPX*’s expression corroborated by TCGA data and previously identified in the differential analysis of combined data. Specifically, the FE fraction uniquely presented four proteins: *MPO*, *IGHV6‐1*, *MASP1*, and *PRSS3*. The pH2 fraction was distinguished by six unique proteins: *ZYX*, *SERPINF2*, *IGLC3*, *TNC*, *IGL1*, and *IGKV3D‐11*. In the pH3 fraction, 27 unique proteins were identified, including *PRG4*, *SERPINC1*, *ITIH2*, *APOC2*, and *F12*, which aligned with the upregulation of the corresponding genes in PCa patients reported in TCGA data; notably, *ITIH2* and *F12* were also identified in the data analysis for combined fractions. Further investigation of these DAPs and their presence in certain EV fractions and upregulation in PCa would be of interest in our future studies.

## 4 DISCUSSION

This research introduces an approach to isolate EVs and fractionate EV sub‐populations from human plasma using SAX fractionation. This approach focuses on the electrostatic properties of EVs and their external surfaces, offering a method designed to reduce contamination in isolates of EVs and EVs sub‐populations from free plasma proteins, a common challenge in EV isolation. Moreover, this approach allowed us to examine charge‐based sub‐populations of EVs, providing insights into the biomolecular compositions that may indicate diverse physiological or pathological conditions of the donors. LC–MS/MS‐based proteomics is one of the core techniques for the molecular characterization of plasma‐derived EVs. The developed method has demonstrated the ability to narrow down the dynamic range of proteins in EV fractions, enabling deeper proteomic profiling. We have also employed orthogonal techniques, such as western blotting, TEM, and NTA, to validate the developed technique and ensure the efficient and robust performance of the method. The described method establishes a careful balance between the purity of EV isolates and EV sub‐populations, the level of depletion of high‐abundance free plasma proteins, and the recovery of EVs and their sub‐populations. Complete depletion of the most predominant free plasma proteins, for example, serum albumin and IgG, without compromising the EV recovery, is challenging and, most probably impossible, given the complexity and high dynamic range of plasma and the heterogeneous nature of EVs. Aggressive free plasma protein depletion techniques could lead to EV loss, particularly because highly abundant plasma proteins can adhere to EV surfaces in what is known as the ‘sponge effect’ (O'Brien et al., 2020), and some of such top abundance proteins may also be incorporated into EV cargo (i.e., becoming EV‐associated and not free plasma proteins). Additionally, lipoproteins are known to interact with certain EV sub‐populations, further complicating the complete depletion of high‐abundance plasma proteins from EV isolates. Our results, which are consistent with previous findings, suggest that high‐abundance plasma proteins are not merely contaminants of EV isolates but may be internal or external tightly associated constituents of specific plasma EV sub‐populations. Thus, the described method was optimized to reduce plasma‐free protein contaminants while preserving the yield of EVs for downstream proteomic analysis.

Furthermore, we have conducted pilot tests to assess the applicability of our method to real‐world clinical samples. We applied this developed method to PCa patient samples and age‐matched controls. The subsequent data analysis identifies DAPs, which could be explored as biomarker candidates for PCa. The study also suggests the association of certain DAPs with EVs based on the reported GO terms and their localization in the cells of origin. This study provides an overview of the possible roles of these proteins in cancer biology, exploring molecular features of EVs and potentially their functions. None of the proteins that we identified as overlapping with those from prior whole‐plasma/serum proteomic PCa studies (Byrne et al., 2009; Larkin et al., 2016), further justifying the pre‐analytical sample processing approach involved in EV enrichment and fractionation by EV surface charge. The developed technique for robust EV fractionation not only enhances the sensitivity but also reveals molecular insights specific to EV biology in PCa, which is challenging to achieve by analysing whole plasma samples. However, the direct correlation between a specific EV sub‐population and the PCa status and progression could not be clearly established in these conducted pilot studies.

While the method shows promising results, it is important to acknowledge its inherent complexity and potential limitations. One key consideration in the development of the methods is the potential impact of technique‐specific biases related to the non‐established availability of an EV markers’ panel. At the initial stages of our study, we mainly relied on CD9 as a marker in western blotting to indicate the presence/relative abundance of EVs in fractions. This approach is more specific than count‐based detection techniques such as NTA or TRPS (tunable resistive pulse sensing). However, while CD9 is widely recognized as an EV membrane protein, its expression can vary significantly among different EV sub‐populations. This variability means that certain EVs with low CD9 expression level and/or those below the detection limit of the technique might not be detected, leading to a potential underestimation of the presence of specific EV populations present in the sample. Such marker‐specific and sensitivity‐related biases highlight the need for a more comprehensive approach that includes multiple markers and advanced detection techniques to capture a broader range of EV sub‐populations.

Another aspect that warrants further discussion is the potential impact of donor material variability on the method's performance. Our initial experiments utilized pooled plasma samples collected in‐house under well‐controlled conditions. Using these samples, we aimed to establish the charge‐based EV fractionation method, ensuring a comprehensive representation of the diverse EV protein content typically found in human plasma. However, the variability inherent in individual donor samples – such as differences in protein concentration, protein composition, EV concentration, and other biological factors – can influence the efficiency, recovery, and specificity of EV isolation methods, which have been shown to affect various EV isolation techniques. Therefore, it is essential to assess how this variability might affect the reproducibility and robustness of our charge‐based fractionation approach when applied to individual donor samples collected at different health, treatment, time of the day, fasting, and other conditions. In addition, intra‐group variability in the proteomic profiles of clinical samples was revealed by PCA and hierarchical clustering, which raises questions about the impact of biological variability and disease heterogeneity on the method's performance. While our initial experiments using pooled healthy donor plasma collected in‐house under well‐controlled conditions demonstrated high reproducibility in the proteomics profiling, the variability in clinical samples suggests that the method's robustness in a clinical setting may be influenced by these factors. Further studies should investigate the consistency of the method across a larger cohort of clinical samples, including different disease stages and sub‐types, to better understand its applicability and limitations.

In summary, the method developed in this study for fractionating plasma‐derived EVs shows the potential to generate new insights into EV biology and the development of diagnostic applications, especially for early detection and monitoring of diseases. With charge‐based isolation of EV sub‐populations, this method offers a promising approach for biomarker discovery and disease mechanism studies. It is worth noting that, although this study serves as a proof‐of‐concept, the method is designed for scalability and reproducibility. With optimization in resin‐to‐plasma ratios and potential adaptation for parallel processing or multi‐well plate formats, this method can feasibly be scaled up for high‐throughput applications, making it suitable for large‐scale biomarker studies where robustness is critical. It is also important to note that the pH‐elution mode used in our protocol may compromise the structural integrity of isolated EVs, although this does not affect our goal in this study, which is focused on molecular characterization of EVs and EV sub‐populations. However, if the structural integrity of the EV sub‐populations is desired, the pH‐based elution steps could be replaced with elution steps based on the increased concentration of salt in the mobile phase of the eluate according to the law of mass action. Still, this adjustment in the elution mode would require additional optimization to ensure effective fractionation. Additionally, while the multi‐step process is necessary to address the complexity of plasma samples, we recognize the potential for future simplification. The development of commercial columns and standardized elution buffers could further streamline the process, enhancing its accessibility and ease of use. Moving forward, we believe that it is essential to advance the developed technique and its applications further using the following considerations.

First, increasing the depth of proteomic profiling of EV fractions by using more advanced LC‐MS and data acquisition techniques, for example, Orbitrap Astral or timsTOF mass spectrometers, and data‐independent acquisition (DIA), which will enable higher throughput analysis while increasing the coverage of proteomic data and/or by using a larger volume of plasma (e.g., 200–300 µL) subjected to EV isolation and fractionation. Moreover, while the available Prostate‐Specific Antigen (PSA)‐based tests are of poor specificity in the detection of PCa, investigating the levels of post‐translational modifications and proteoforms of such proteins as PSA and prostate‐specific membrane antigen (PSMA) in a targeted manner in plasma, in addition to the described here EV sub‐population‐based profiling, may provide more specificity and sensitivity in diagnostic and prognostic applications. Additionally, a combination of proteomic and transcriptomic readouts, for example, quantitative profiling of extracellular RNA and targeted analysis of specific markers, including PCA3, a non‐coding RNA known for its high specificity in PCa diagnosis, is proposed in further investigations.

Next, using a larger and better‐controlled sample set that would include a comprehensive set of metadata associated with the disease and treatment states, sample collection, fasting status, and other relevant conditions will help enhance the statistical significance and rigour of future experiments.

Future endeavours are considered to enable the multi‐omic analysis of EVs and their sub‐populations, including proteomic, glycomic, lipidomic, transcriptomic, and genomic aspects. This comprehensive approach is expected to confirm the efficiency of EV fractionation techniques and shed light on molecular mechanisms and functions orchestrated and conducted by EVs. In addition, a further expansion of the liquid biopsy‐based biomarker discovery framework to include a broader range of diseases and physiological states is another logical step for future applications of the developed technique. The methodologies developed in this study have the potential to go beyond PCa, thus providing a strategy for enriching markers in pan‐cancer diagnostic applications and in the diagnostics of other pathologies. This extension is a logical transition to a more comprehensive approach to studying disease molecular manifestations and mechanisms more precisely, which can be enabled and enhanced by the described EV fractionation technique.

## AUTHOR CONTRIBUTIONS

**Xianyi Su**: Conceptualization (lead); data curation (lead); formal analysis (lead); investigation (lead); methodology (lead); project administration (lead); visualization (lead); writing—original draft (lead); writing—review and editing (lead). **Getúlio Pereira de Oliveira Júnior**: Data curation (supporting); writing—review and editing (supporting). **Anne‐Lise Marie**: Writing—review and editing (supporting). **Michal Gregus**: Writing—review and editing (supporting). **Amanda Figueroa‐Navedo**: Writing—review and editing (supporting). **Ionita C. Ghiran**: Funding acquisition (supporting); resources (supporting); writing—review and editing (supporting). **Alexander R. Ivanov**: Conceptualization (lead); data curation (lead); funding acquisition (lead); investigation (lead); methodology (lead); project administration (lead); resources (lead); supervision (lead); writing—review and editing (lead).

## CONFLICT OF INTEREST STATEMENT

The authors declare no conflicts of interest.

## Supporting information



Supporting Information
