# Supplementary material for: Enhanced proteomic profiling of human plasma‐derived extracellular vesicles through charge‐based fractionation to advance biomarker discovery potential
Source: J Extracell Vesicles. 2024 Dec 6;13(12):e70024. doi: 10.1002/jev2.70024 (PMC11621968; doi:10.1002/jev2.70024)
Supplement: Supplementary file 1 — Supporting Information [file JEV2-13-e70024-s001.docx]

## **Supporting Information**

Enhanced Proteomic Profiling of Human Plasma-Derived Extracellular Vesicles through Charge-Based Fractionation to Advance Biomarker Discovery Potential

Xianyi Su ${}^{1}$, Getúlio Pereira de Oliveira Júnior${}^{1}$, Anne-Lise Marie${}^{1}$, Michal Gregus${}^{1}$, Amanda Figueroa-Navedo${}^{1}$, Ionita C. Ghiran${}^{2}$, and Alexander R. Ivanov ${}^{1,*}$

${}^{1}$ Department of Chemistry and Chemical Biology, Barnett Institute of Chemical and Biological Analysis, Northeastern University, Boston, Massachusetts, USA

${}^{2}$ Department of Anesthesia, Beth Israel Deaconess Medical Center, Harvard Medical School, Boston, Massachusetts, USA

${}^{*}$Corresponding author: E-mail [a.ivanov@northeastern.edu](mailto:a.ivanov@northeastern.edu)

**Table of Contents**

Experimental Techniques (Materials, EV Characterization, Nanoflow LC-MS/MS Proteomic Profiling, Data Analysis) Pages 2 - 5

Supplementary Figures 1 - 7 Pages 6 - 12

Supplementary Tables 1 - 4 Pages 13 - 16

References Pages 16

## Materials

2-Iodoacetamide (IAA), acetic acid, ammonium bicarbonate, bovine serum albumin (BSA), and urea were purchased from Sigma-Aldrich. Capto Core 700 (CC700) resin, Dulbecco’s Phosphate-Buffered Saline (dPBS), and Q-Sepharose resin were procured from Cytiva. Acetonitrile (ACN), formic acid (FA), Tris(2-carboxyethyl) phosphine (TCEP), thiourea, skim milk powder, 20x PBS Tween™ 20 (PBST) buffer, SuperSignal West Femto maximum sensitivity substrate, Parafilm, and 5mL centrifuge columns were obtained from Thermo Fisher Scientific. 4x Lithium dodecyl sulfate (LDS) sample buffer, 10x dithiothreitol (DTT), 4-12% Bis-Tris gel, and polyvinylidene difluoride (PVDF) membrane were purchased from Invitrogen. Amicon Ultra 10 kDa MWCO filter was purchased from MilliporeSigma. C18 membrane disk was acquired from CDS. A carbon film-supported copper gilder grid was obtained from Electron Microscopy Sciences. ReproSil-Pur 120 C18-AQ beads were purchased from Dr. Maisch. Trypsin/Lys-C mix was obtained from Promega. Anti-albumin (sc-271605), anti-apoB-100 (sc-13538), anti-IgG (sc-69786) antibodies, mouse anti-rabbit IgG-HRP secondary antibody (sc-2357), and HRP-conjugated mouse IgG light chain binding protein (sc-516102) were all purchased from Santa Cruz. Anti-CD9 (10626D), anti-integrin β1 antibodies (14-0299-82), and 10 nm colloidal gold-conjugated goat anti-mouse IgG secondary antibody (A-31561) were sourced from Invitrogen. Anti-Rap-1b (10840-1-AP) antibody was obtained from Proteintech.

## EV Characterization

### 1D-Polyacrylamide Gel Electrophoresis (1D-PAGE)

Starting sample ST and all fractions (15 µL plasma input equivalent) were concentrated using a FreeZone lyophilizer (Labconco, US), then mixed with 5 µL of 4x LDS sample buffer and 2 µL of 10x DTT for a total volume of 20 µL. This mixture was then heated at 70 °C for 10 min for lysis and denaturation. Electrophoresis was conducted on a 4-12% Bis-Tris gel at 200 V for 1 h.

### Western Blotting

Western blot analysis was performed on six proteins, categorized into three high-abundance plasma species (albumin, immunoglobulin G (IgG), and apolipoprotein B-100 (apoB-100)) and three EV-related proteins (CD9-antigen, integrin β1, and Ras-related protein Rap-1b). Each protein underwent individual blotting due to their significantly different abundances. The sample volumes, in terms of the plasma input, were as follows: 10 µL for albumin, 20 µL each for IgG and apoB-100, 50 µL each for CD9 and Rap-1b, and 100 µL for integrin β1. After performing the same lysis, denaturation, and electrophoresis protocols as in 1D-PAGE, proteins were transferred to a PVDF membrane using a Trans-Blot Turbo Transfer System (Bio-Rad, US). Distinctly, CD9 detection was performed under non-reducing conditions throughout the entire process to maintain epitope reactivity. After transfer, membranes were blocked for 1 h at room temperature with 5% skim milk in 1x PBST buffer, then incubated overnight at 4 °C with primary antibodies: anti-human albumin (1:500), IgG (1:1,000), apoB-100 (1:500), CD9 (1:750), integrin β1 (1:250), and Rap-1b (1:500). Subsequently, membranes were treated with HRP-conjugated mouse IgG𝜅 light chain-binding protein (1:2,000), or mouse anti-rabbit IgG-HRP secondary antibody (1:1,000) for Rap-1b blotting, at room temperature for 1 h. Chemiluminescent signals were then activated using SuperSignal West Femto maximum sensitivity substrate, and the images were documented by a ChemiDoc MP Imaging System (Bio-Rad, US). Quantitative optical densitometry analysis of the bands detected in western blot experiments was performed using Image Lab (v6.1) software (Bio-Rad, US).

### Transmission Electron Microscopy Imaging

The TEM protocol for EV imaging began with a concentration step to increase the EV particle quantity. All samples, equivalent to a 50 µL plasma input volume, were concentrated to a final 20 μL volume using an Amicon Ultra 10 kDa MWCO filter in a microcentrifuge (Eppendorf, Germany) at 14,000 x *g* for 30 min at 4 °C. Subsequently, 5 µL of each concentrated sample was carefully placed onto a parafilm sheet. On the top of each sample-containing droplet, a glow-discharged 10 nm-thick carbon film-supported copper gilder grid was positioned for an incubation period of 15 min at room temperature. After incubation, grids were gently rinsed with water to remove any unattached or excess samples. For enhanced contrast, samples underwent negative staining using a 2% uranyl acetate solution for 2 min.

Additionally, to increase specificity, immunogold TEM was performed on EV-enriched samples (i.e., ST, pH2, and FE). This involved initially blocking the sample-loaded grids with 1% BSA for 10 min. The grids were then incubated for 30 min on a 5 µL drop of anti-human CD9 primary antibody diluted (1:15) in 1% BSA. This was followed by three 10-min washes with 1x dPBS. The grids were then incubated for 20 min with droplets of 10 nm colloidal gold-conjugated secondary antibody (1:30) in 1% BSA, and subsequently washed twice with 1x dPBS for 5 min each plus four times with water for 10 min each. Finally, the prepared samples were visualized using a JEM 1010 TEM microscope (JEOL Ltd., Japan) equipped with a 2k x 2k pixels AMT XR-41B CCD camera system.

### Nanoparticle Tracking Analysis

NTA was conducted using a ZetaView instrument (Particle Metrix, Germany). Before the analysis, each sample was diluted 50 times to a final volume of 1 mL and then thoroughly vortexed to ensure homogeneity. This dilution step is crucial to avoid detector saturation with overabundant signals and achieve accurate measurements. During the analysis, the selected laser wavelength was 488 nm, and the filter was set to detect scattered light. The sample chamber of the instrument was maintained at room temperature. For measuring particle size distribution, the procedure included two cycles, each consisting of 11 different positions within the sample chamber. For zeta potential measurements, five cycles were conducted where two stationary layers were established at relative positions of 0.149 and 0.851 within the chamber to ensure accurate measurements.

### Nanoflow LC-MS/MS Proteomic Profiling

#### Sample Preparation

The sample lysis/digestion preparation was performed with an optimized OmSET protocol^1^ with minor changes to minimize sample loss. Specifically, 150 µL (plasma input volume equivalent) of each EV sample was introduced into a 200 µL pipet tip. This tip was pre-packed with four layers of a C18 membrane obtained using a blunt tip needle of 14-gauge. Lysis was performed for 20 min at room temperature using a mixture composed of 8 M urea, 2.5 M thiourea, and 6 mM TCEP in 25 mM ammonium bicarbonate at pH 8. This was followed by a simultaneous reduction and alkylation step with 25 mM TCEP and 10 mM IAA in 25 mM ammonium bicarbonate (pH 8) for 45 min in the dark at room temperature. Overnight proteolytic digestion was then executed at 45 °C using trypsin and Lys-C mix at a 1:10 enzyme-to-substrate ratio (for each enzyme). The digested peptides were subsequently eluted into glass LC inserts with three successive 10 µL aliquots of a solution of 65% ACN and 0.1% FA. These samples were then lyophilized to complete dryness and stored at -80 °C. Prior to LC injection, they were reconstituted in a 5 µL solution of 1% ACN with 0.1% FA.

#### Nanoflow LC Conditions

The nanoflow LC (nLC) separation of digested peptides employed an in-house packed C18 column. A fused silica capillary (75 μm ID x 360 μm OD) was laser-pulled to produce an electrospray ionization (ESI) emitter tip. The pulled capillary was carefully packed with ReproSil-Pur 120 C18-AQ beads with a mean diameter of 1.9 μm and pore size of 120 Å. The length of the packed column was 15 cm.

For method development with healthy donor samples, we utilized the Ultimate 3000 nLC system (Thermo Fisher Scientific, US). The analytical column was connected via a tee union to a nanoViper transfer line (20 μm ID x 360 μm OD x 1 m length) linked to the LC switching valve. The ESI voltage was applied at the tee union for ionization of analytes during elution. The pilot tests with clinical samples employed the Vanquish Neo UHPLC system (Thermo Fisher Scientific, US). In both setups, the column was housed in a pencil column heater (Phoenix S&T, US) and maintained at 60 °C to ensure consistent retention times and performance.

Chromatographic conditions involved a mobile phase A of 0.1% FA in water, while mobile phase B comprised 0.1% FA in ACN. With the Ultimate 3000 system, samples were loaded onto the column at 350 nL/min for 20 min, followed by an elution at 120 nL/min over a 120-min gradient from 1% B to 25% B. On the Vanquish Neo, the gradient was shortened to 45 min at 200 nL/min.

#### Nanoflow LC-Tandem Mass Spectrometry (MS/MS)

For method development, each sample was subjected to triplicate (equivalent to 45 μL plasma input for one injection) nLC-MS/MS analysis employing an Exploris 480 Orbitrap mass spectrometer (Thermo Fisher Scientific, US). The ESI voltage was set at 1.8 kV using an EASY-Spray ionization source (Thermo Fisher Scientific, US), and the ion transfer tube temperature was held at 275 °C. The system was set to operate in positive ESI and data-dependent acquisition (DDA) modes. For full MS^1^ scans, the spectral range was 375-1,600 *m/z*, and the resolution was 120,000 (at 200 *m/z*). Specific adjustments included a normalized automatic gain control (AGC) target at 300%, the maximum injection time on auto mode, microscans at a value of 1, and the RF lens intensity set to 50%. For MS^2^ analysis, higher-energy collisional dissociation (HCD) was maintained at a normalized energy of 30%. Precursor ions were selected for fragmentation at a Top Speed mode, where 3 sec lasted between two master scans, targeting ions with charge states between 2 and 6 and exhibiting a minimum intensity of 5xE3. To mitigate redundancy in precursor ion selection, a dynamic exclusion of 45 sec and an isotope exclusion were used. MS^2^ spectra were acquired at a resolution of 30,000 (at 200 *m/z*) with a defined isolation window of 2 *m/z*. Other settings included a standard AGC target, an auto-regulated maximum injection time, and microscans set to 1. The “define first mass” mode was selected and set to start with 110 *m/z*.

A similar analytical procedure was employed for clinical samples, except that samples were injected in duplicates (equivalent to 70 μL plasma input). ESI voltage was increased to 2 kV. The MS^2^ resolution was heightened to 60,000 (at 200 *m/z*), and the isolation window was set at 3 *m/z*.

#### Data Analysis

Acquired raw files were processed using the Proteome Discoverer software (v. 3.0, Thermo Fisher Scientific). These files were searched against the UniProtKB/SwissProt human database (Release 2020.01). For method development, the Sequest HT search engine was employed. This analysis operated with a mass tolerance of 5 ppm for precursor ions and 0.02 Da for fragment ions. For the clinical samples, an AI-based engine, CHIMERYS, was utilized for the spectral search using a fragment mass tolerance of 5 ppm. Both searches allowed for up to two missed cleavage sites per peptide, with the minimum peptide length set to seven amino acid residues. Carbamidomethylation of cysteine residues was selected as a static modification. Spectrum matching further benefited from INFERYS rescoring under automated mode. To ensure data reliability, a stringent false discovery rate (FDR) threshold of 1% was applied at both the peptide and protein levels. Quantitative analysis of the identified proteins was executed through a label-free quantification (LFQ) approach. This process leveraged unique peptides with their respective spectrum abundances. The mass spectrometry proteomics data have been deposited to the ProteomeXchange Consortium via the PRIDE^2^ partner repository with the dataset identifier PXD049702.

Further data analysis and visualization for intercorrelation analysis, principal component analysis (PCA), Venn diagrams, differential analysis (PCa vs. healthy control samples), volcano plots, and Sankey diagrams were performed within R. Hierarchical clustering heatmaps were generated with the open-access TBtools-II (v1.120) software^3^ and R. KEGG (Kyoto Encyclopedia of Genes and Genomes) annotation was performed with DAVID Knowledgebase^4^. GO (Gene Ontology) enrichment was conducted in FunRich (v3.1.3)^5^.


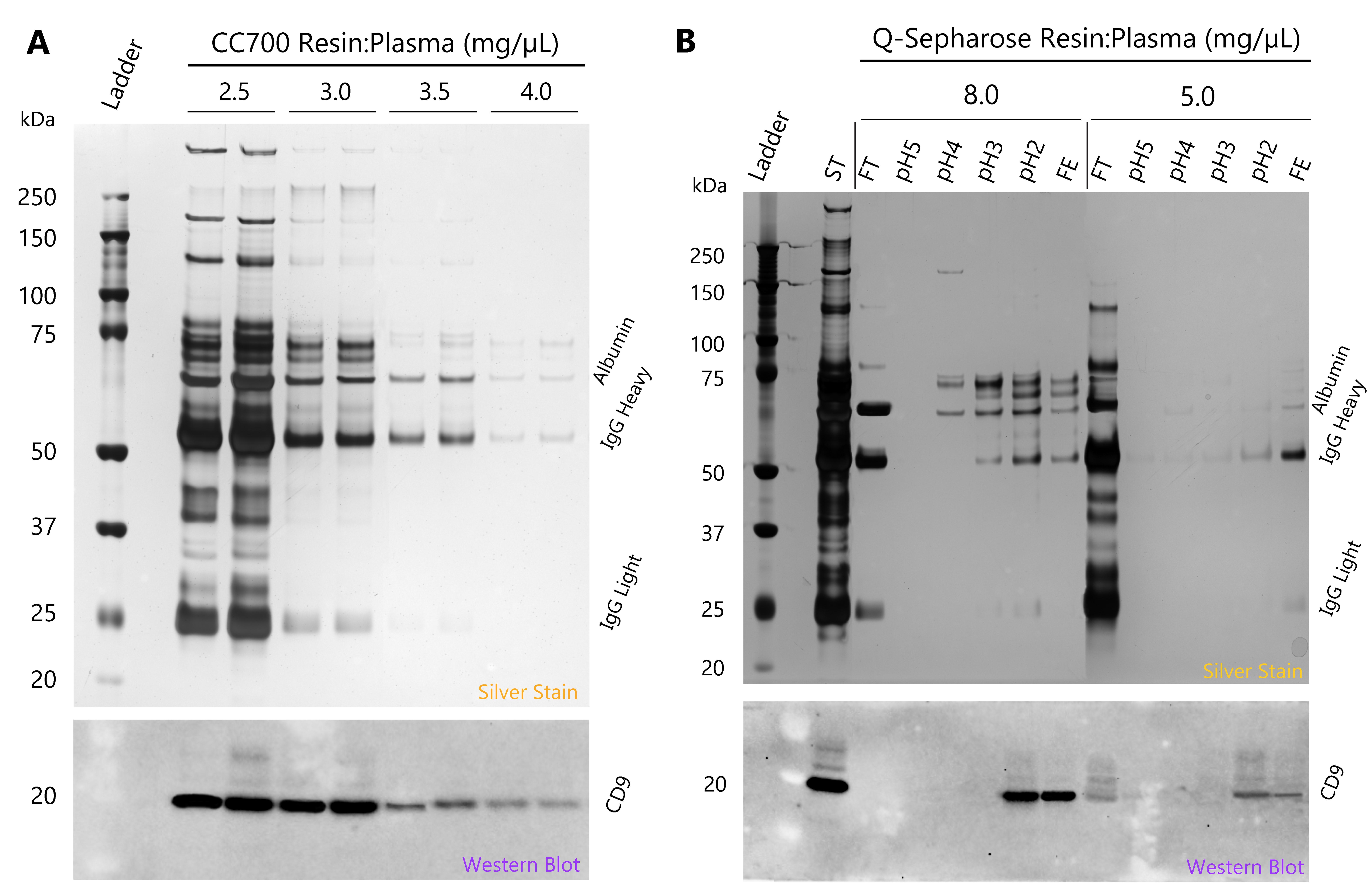


**Supplementary Figure 1. Representative Examples of Results Acquired during Method Development Focused on Balancing Sample Purity and EV Recovery Rate**. (A) Capto Core 700 Pre-Purification: examples of four Capto Core 700 (CC700) resin to plasma ratios (mg/μL) - 2.5, 3.0, 3.5, and 4.0 that were evaluated in duplicates using SDS-PAGE silver staining (top panel) and anti-CD9 western blotting (bottom panel). The SDS-PAGE gel allows the identification of major plasma proteins, including albumin (~67 kDa MW) and IgG (~50 kDa heavy chain and ~25 kDa light chain) based on their migration relative to the protein ladder. Concurrently, specific CD9 bands are identified on the western blot membrane. A ratio of 3.0 was determined to offer the best compromise between purity and EV recovery rate. (B) Q-Sepharose SAX Fractionation: at this stage, resin to plasma ratios of 8.0 and 5.0 were examined in the shown examples. The anti-CD9 western blot (bottom panel) demonstrated enhanced EV recovery, and the SDS-PAGE gel (top panel) showed a clearer fractionation pattern of plasma proteins, indicating that a ratio of 8.0 is appropriate for reaching the desired performance. In both (A) and (B), the SDS-PAGE sample loading volume in each gel lane corresponds to 15 μL of input plasma. For the anti-CD9 western blot, the loading volume of 50 μL of input plasma was used.


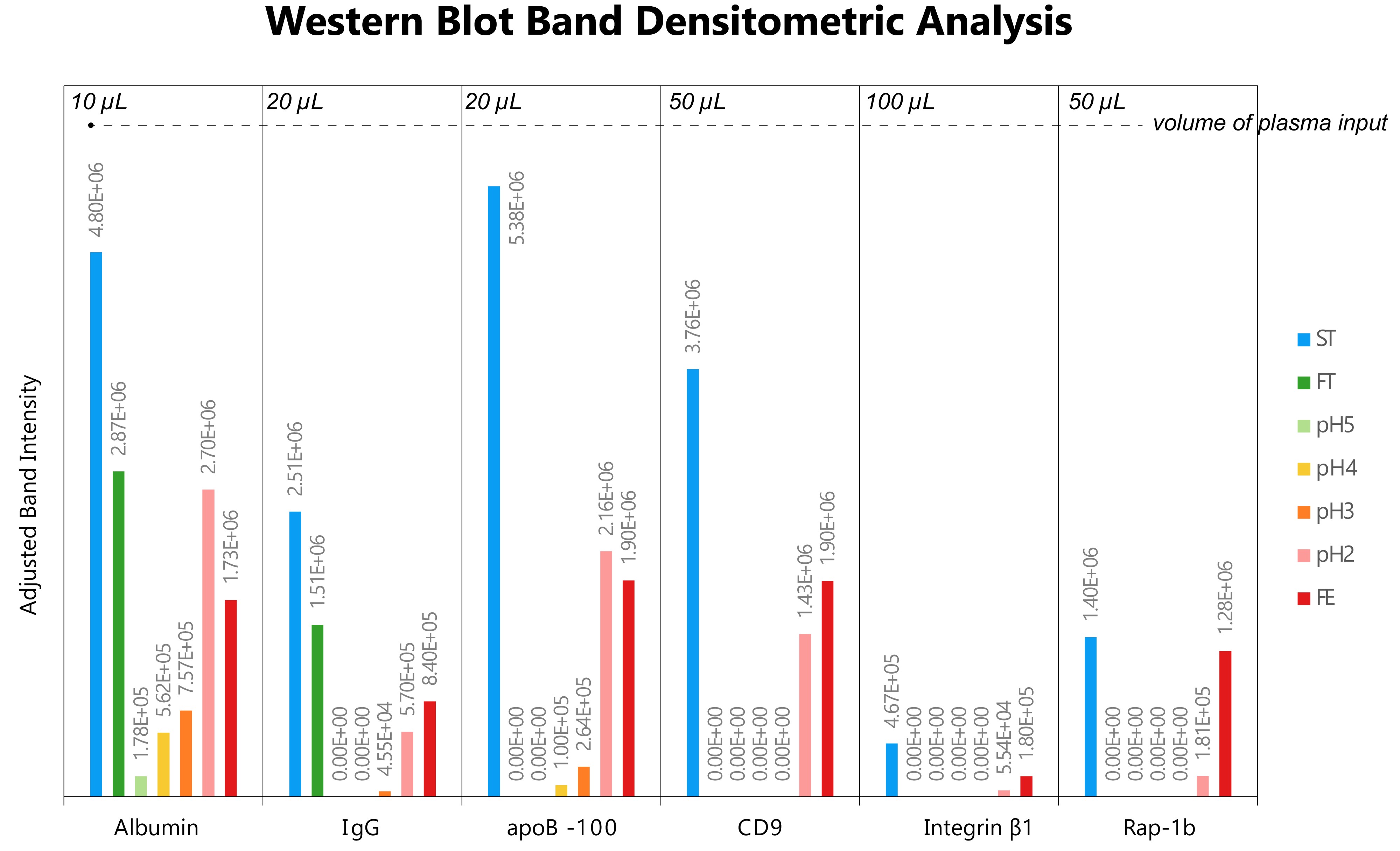


**Supplementary Figure 2. Representative Results for Optical Densitometry Analysis of Western Blot Band Intensities.** The italicized text above each lane indicates the sample loading volume corresponding to the plasma input.


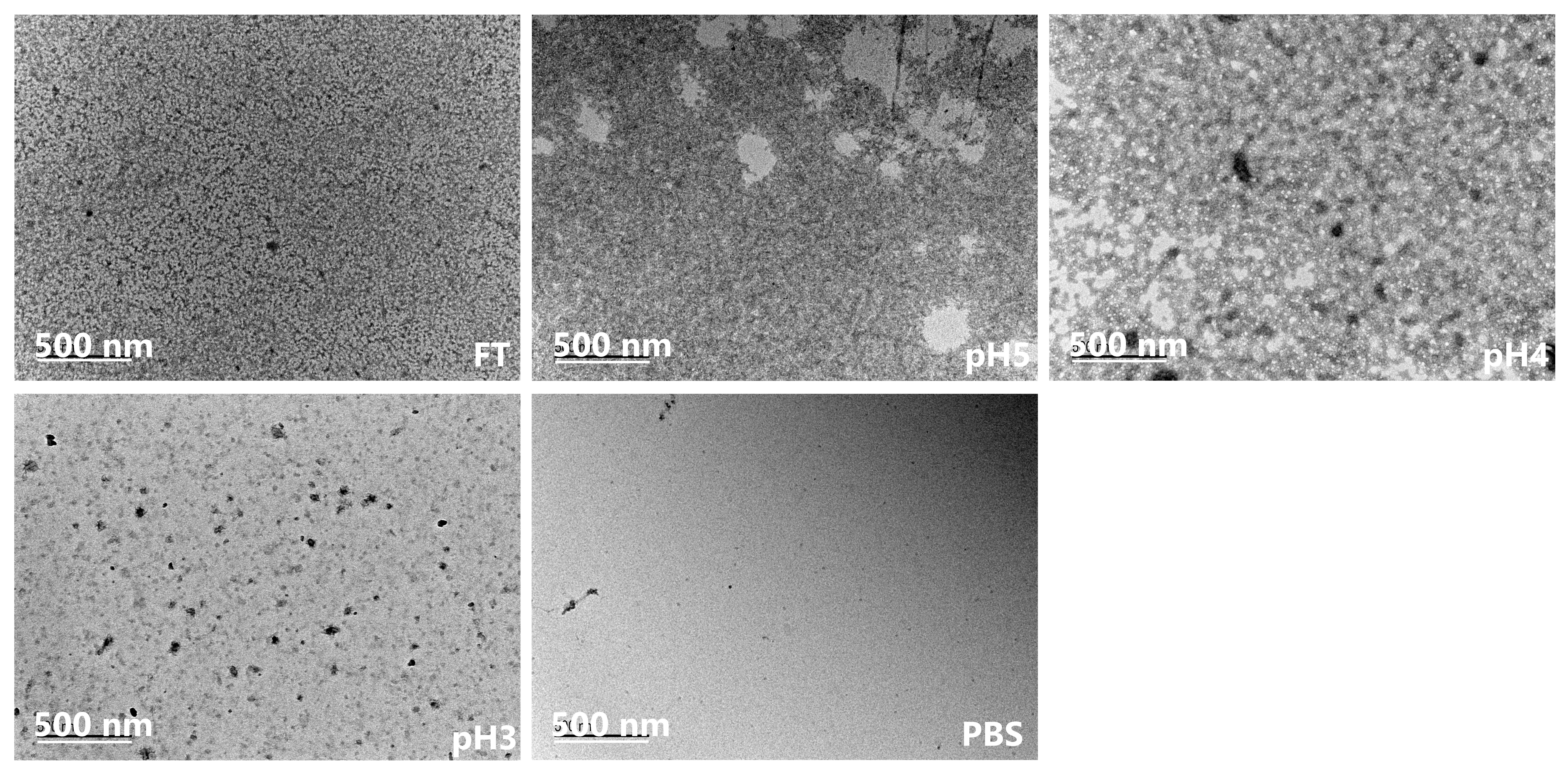


**Supplementary Figure 3. Representative TEM Images for the FT, pH5, pH4, and pH3 Fractions and a Negative Control of a PBS Blank.** No prominent vesicles or particles could be identified in these samples.


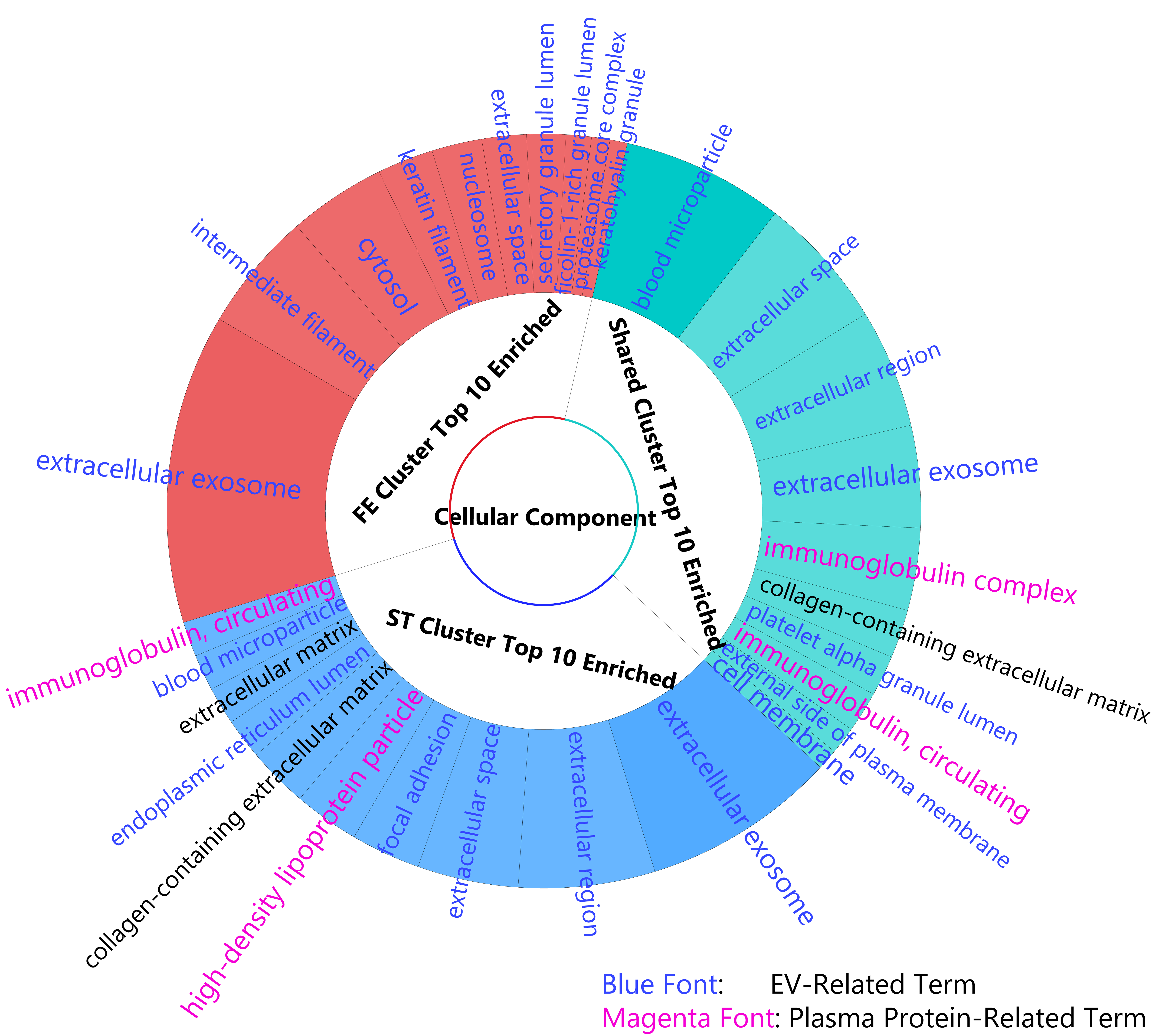


**Supplementary Figure 4. The Results of GO Enrichment Analysis for the Clusters Shown in Figure 3C (Full Version).**


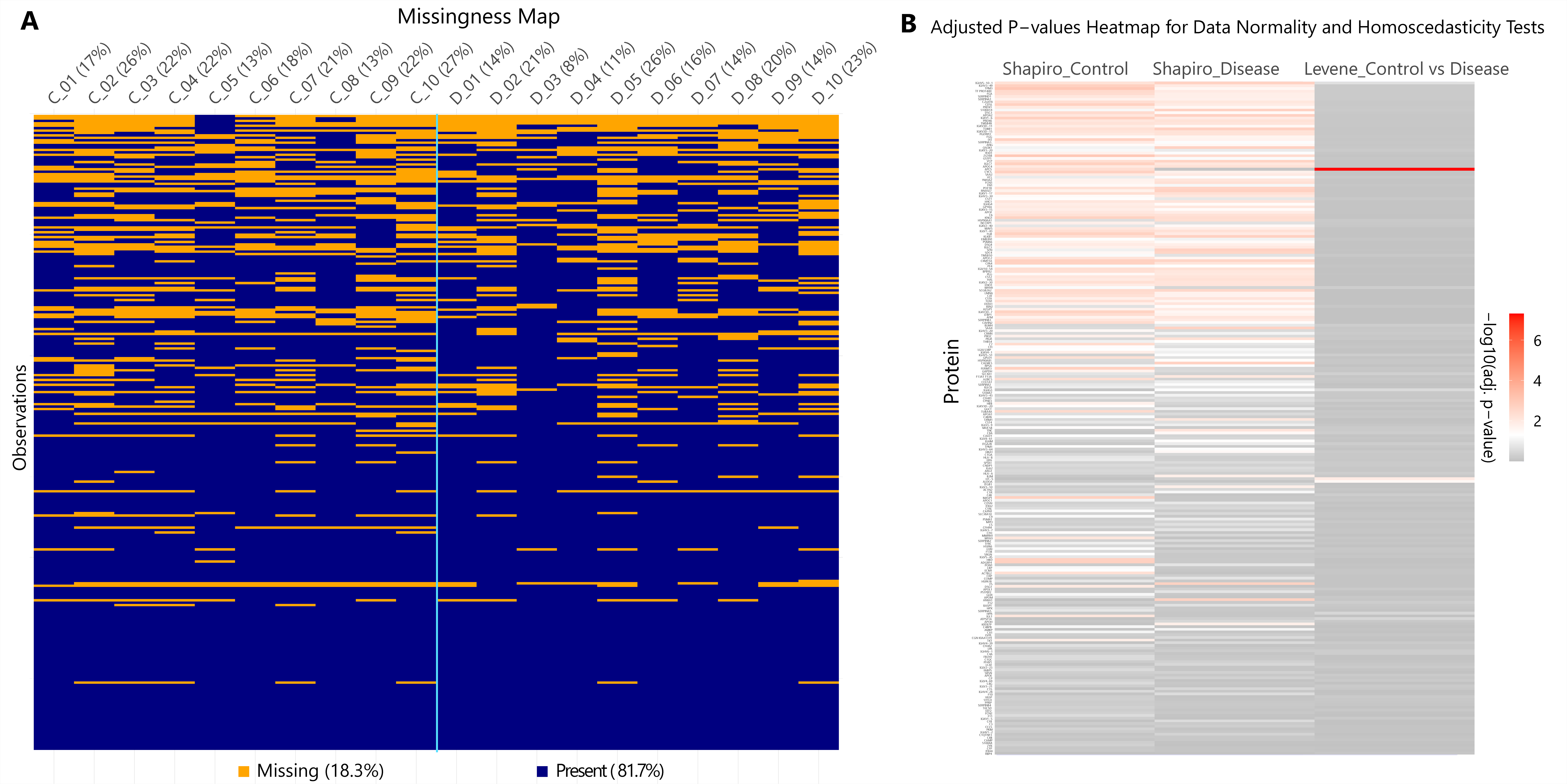


**Supplementary Figure 5. Proteomics Data Evaluation for the Disease and Control Samples.** (A) Data missingness map for the acquired proteomic data shows an average missing rate of 18.3%. (B) Assessment of data normality of individual groups and homoscedasticity between them, using the Shapiro-Wilk and Levene’s tests, respectively. An adjusted *p*-value < 0.05 indicates deviations from normality or homoscedasticity.


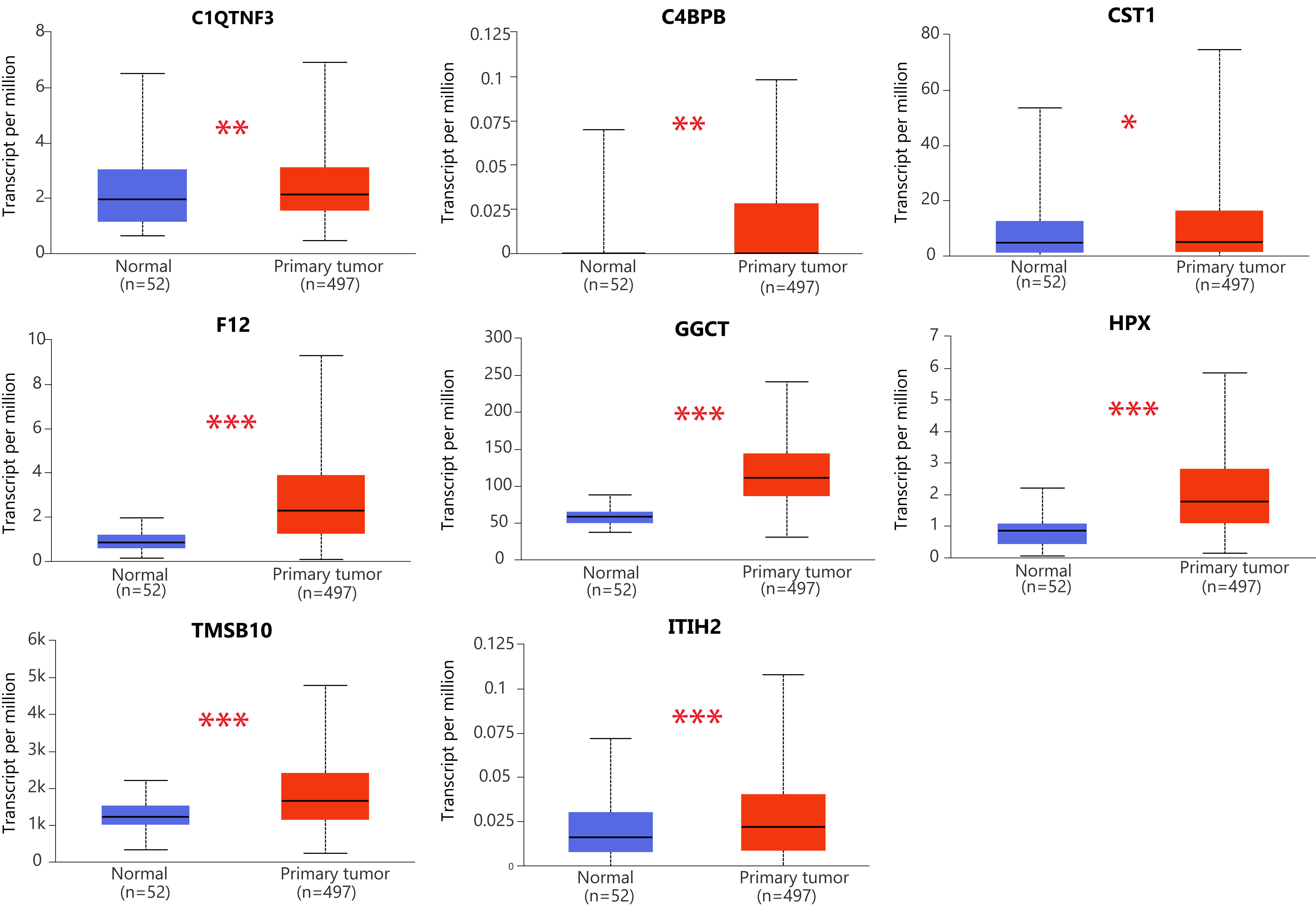


**Supplementary Figure 6. Quantitative Transcriptomic Profiles for Selected Genes Corresponding to the Detected Eight DAPs Based on the TCGA Gene Expression Data in PRAD (Prostate Adenocarcinoma; TCGA-PRAD).** Adapted from the original figures on UALCAN portal^6, 7^.


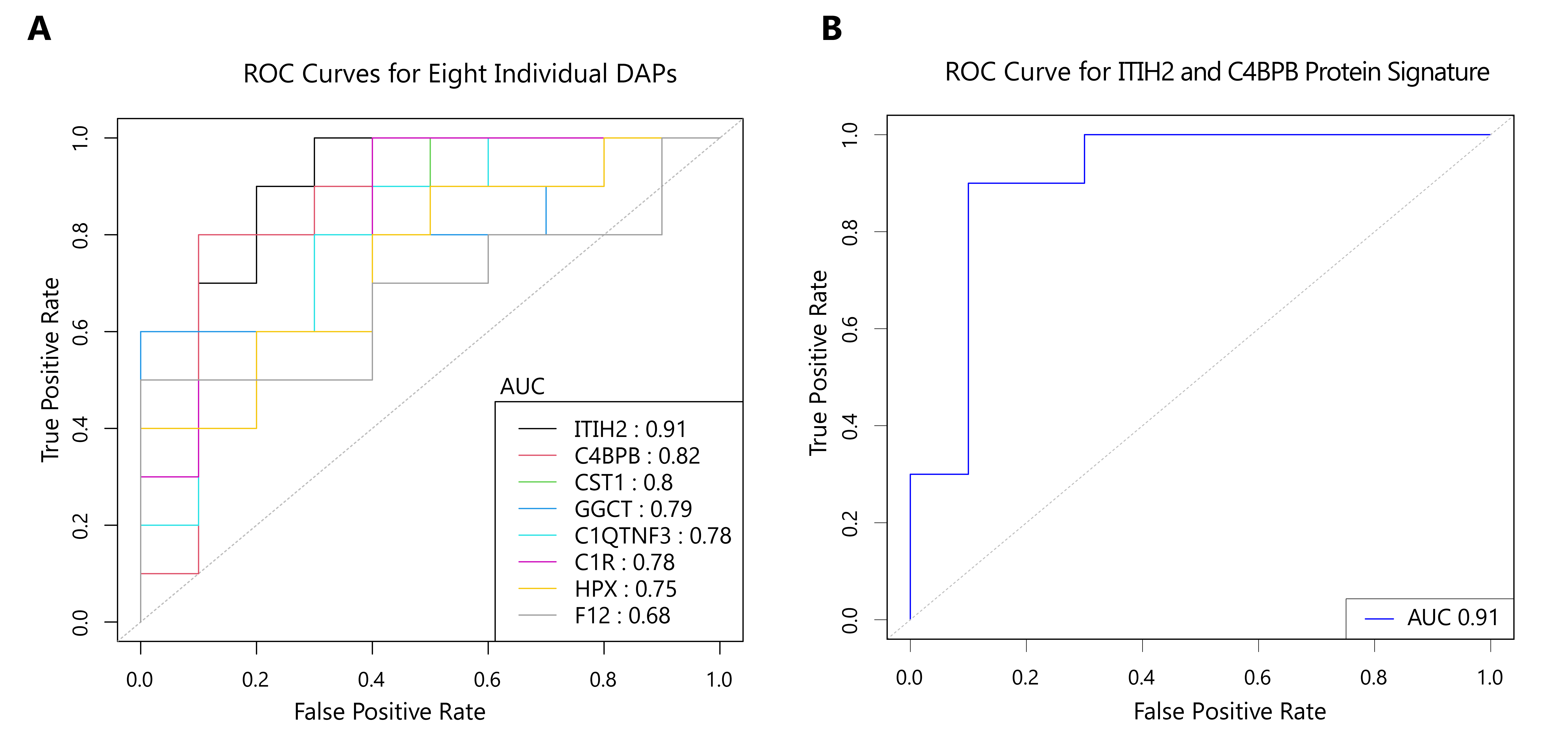


**Supplementary Figure 7. Receiver Operating Characteristic (ROC) Curves for Selected Eight DAPs between PCa and Control Groups.** *ITIH2* demonstrates excellent diagnostic performance based on the quantitative proteomics data (AUC = 0.91). *C4BPB* and *CST1* show AUCs of 0.82 and 0.80, respectively. *GGCT*, *C1QTNF3*, *C1R*, and *HPX* exhibit AUCs ranging from 0.75 to 0.79. *F12* indicates a lower discriminative ability with an AUC of 0.68. (B) The ROC curve for the *ITIH2* and *C4BPB* developed based on the quantitative proteomic profiles demonstrates high diagnostic accuracy and sensitivity with an AUC of 0.91, indicating the potential for distinguishing disease states with this combined signature set.

**Supplementary Table 1: Gene Names of Proteins**

| *Gene* | Protein |
| --- | --- |
| *A2M* | Alpha-2-Macroglobulin |
| *ACAP1* | ArfGAP with coiled-coil, ankyrin repeat, and PH domains 1 |
| *ADGRF4* | Adhesion G protein-coupled receptor F4 |
| *ALB* | Albumin |
| *ALDOA* | Aldolase A |
| *APOA1* | Apolipoprotein A-I |
| *APOB* | Apolipoprotein B |
| *APOC2* | Apolipoprotein C-II |
| *ATP5F1A* | ATP synthase F1 subunit alpha |
| *BASP1* | Brain abundant membrane attached signal protein 1 |
| *C1QTNF3* | C1q and TNF related 3 |
| *C3* | Complement C3 |
| *C4B* | Complement C4B |
| *C4BPB* | C4b-binding protein beta chain |
| *C5* | Complement C5 |
| *CAPN1* | Calpain-1 |
| *CASP3* | Caspase-3 |
| *CDSN* | Corneodesmosin |
| *CPA4* | Carboxypeptidase A4 |
| *CST1* | Cystatin SN |
| *CSTA* | Cystatin A |
| *EMILIN1* | Elastin microfibril interfacer 1 |
| *F10* | Coagulation factor X |
| *F12* | Coagulation factor XII |
| *GAPDH* | Glyceraldehyde-3-phosphate dehydrogenase |
| *GGCT* | Gamma-glutamylcyclotransferase |
| *GP1BA* | Glycoprotein Ib alpha chain |
| *H2BC5* | Histone cluster 2 H2B family member c |
| *HPX* | Hemopexin |
| *HSPA6* | Heat shock 70 kDa protein 6 |
| *IGHG* | Immunoglobulin heavy constant gamma |
| *IGHV6-1* | Immunoglobulin heavy variable 6-1 |
| *IGKV3D-11* | Immunoglobulin kappa variable 3D-11 |
| *IGL1* | Immunoglobulin lambda-1 light chain |
| *IGLC3* | Immunoglobulin lambda constant 3 |
| *ITGA2B* | Integrin alpha-IIb |
| *ITIH2* | Inter-alpha-trypsin inhibitor heavy chain H2 |
| *MASP1* | Mannan-binding lectin serine protease 1 |
| *MPO* | Myeloperoxidase |
| *MUC2* | Mucin 2 |
| *MUC5B* | Mucin 5B |
| *MUC6* | Mucin 6 |
| *PF4V* | Platelet factor 4 variant |
| *PON1* | Paraoxonase 1 |
| *PRG4* | Proteoglycan 4 |
| *PRSS3* | Serine protease 3 |
| *RAB10* | Ras-related protein Rab-10 |
| *RAB11B* | Ras-related protein Rab-11B |
| *RAB14* | Ras-related protein Rab-14 |
| *RAB27B* | Ras-related protein Rab-27B |
| *SERPINC1* | Serpin family C member 1 |
| *SERPINF2* | Serpin family F member 2 |
| *SERPING1* | Serpin family G member 1 |
| *SNAP23* | Synaptosomal-associated protein 23 |
| *SRGN* | Serglycin |
| *TMSB10* | Thymosin beta-10 |
| *TMSB4X* | Thymosin beta-4, X-linked |
| *TNC* | Tenascin-C |
| *VASP* | Vasodilator-stimulated phosphoprotein |
| *YWHAZ* | Tyrosine 3-monooxygenase/tryptophan 5-monooxygenase activation protein zeta |
| *ZYX* | Zyxin |

*Based on www.uniprot.org*

**Supplementary Table 2: Clinical Sample Information**

| Age Group | Prostate Cancer Patients | | | Controls | | |
| --- | --- | --- | --- | --- | --- | --- |
|  | *Cat. #* | *Age* | *In-House Label* | *Cat. #* | *Age* | *In-House Label* |
| 1 | 991-58-S-PC | 47 | D_01 | 991-58-PS | 46 | C_01 |
| 2 | 991-58-S-PC | 58 | D_02 | 991-58-PS | 58 | C_02 |
| 3 | 991-58-S-PC | 60 | D_03 | 991-58-PS | 63 | C_03 |
| 4 | 991-58-S-PC | 65 | D_04 | 991-58-PS | 66 | C_04 |
| 5 | 991-58-S-PC | 69 | D_05 | 991-58-PS | 71 | C_05 |
| 6 | 991-58-S-PC | 70 | D_06 | 991-58-PS | 72 | C_06 |
| 7 | 991-58-S-PC | 74 | D_07 | 991-58-PS | 75 | C_07 |
| 8 | 991-58-S-PC | 74 | D_08 | 991-58-PS | 75 | C_08 |
| 9 | 991-58-S-PC | 76 | D_09 | 991-58-PS | 76 | C_09 |
| 10 | 991-58-S-PC | 88 | D_10 | 991-58-PS | 86 | C_10 |

*www.leebio.com*

**Supplementary Table 3: Eluting Buffer Composition**

| Eluting Buffer | 0.3% Acetic Acid | 50mM Ammonium Acetate | 2.5% Acetic Acid | 1.5% Formic Acid | pH |
| --- | --- | --- | --- | --- | --- |
| pH 5 | 10% | 90% | - | - | 5.44 |
| pH 4 | 50% | 50% | - | - | 4.54 |
| pH 3 | 95% | 5% | - | - | 3.41 |
| pH 2 | - | - | 100% | - | 2.56 |
| FE (Final Elution) | - | - | - | 100% | 2.02 |

**Supplementary Table 4: The Number of Identified and LFQ Quantitated Protein in Each Fraction for Evaluated Clinical Samples**

| Age Group | Control | | | Disease | | |
| --- | --- | --- | --- | --- | --- | --- |
|  | *FE* | *pH2* | *pH3* | *FE* | *pH2* | *pH3* |
| 1 | 194 | 191 | 195 | 211 | 213 | 204 |
| 2 | 170 | 169 | 167 | 185 | 191 | 181 |
| 3 | 192 | 184 | 170 | 210 | 222 | 206 |
| 4 | 182 | 186 | 169 | 206 | 197 | 197 |
| 5 | 216 | 197 | 182 | 173 | 173 | 169 |
| 6 | 182 | 207 | 185 | 193 | 192 | 182 |
| 7 | 174 | 179 | 172 | 204 | 205 | 187 |
| 8 | 186 | 209 | 198 | 184 | 179 | 178 |
| 9 | 180 | 186 | 181 | 205 | 217 | 199 |
| 10 | 174 | 176 | 163 | 191 | 177 | 174 |

**References**

1. Kostas, J.C., Greguš, M., Schejbal, J., Ray, S. & Ivanov, A.R. Simple and Efficient Microsolid-Phase Extraction Tip-Based Sample Preparation Workflow to Enable Sensitive Proteomic Profiling of Limited Samples (200 to 10,000 Cells). *Journal of Proteome Research* **20**, 1676-1688 (2021).

2. Perez-Riverol, Y. et al. The PRIDE database resources in 2022: a hub for mass spectrometry-based proteomics evidences. *Nucleic Acids Research* **50**, D543-D552 (2022).

3. Chen, C. et al. TBtools-II: A “one for all, all for one” bioinformatics platform for biological big-data mining. *Molecular Plant* **16**, 1733-1742 (2023).

4. Sherman, B.T. et al. DAVID: a web server for functional enrichment analysis and functional annotation of gene lists (2021 update). *Nucleic Acids Research* **50**, W216-W221 (2022).

5. Fonseka, P., Pathan, M., Chitti, S.V., Kang, T. & Mathivanan, S. FunRich enables enrichment analysis of OMICs datasets. *Journal of Molecular Biology* **433**, 166747 (2021).

6. Chandrashekar, D.S. et al. UALCAN: A Portal for Facilitating Tumor Subgroup Gene Expression and Survival Analyses. *Neoplasia* **19**, 649-658 (2017).

7. Chandrashekar, D.S. et al. UALCAN: An update to the integrated cancer data analysis platform. *Neoplasia (New York, N.Y.)* **25**, 18-27 (2022).
